# Between-species differences in gene copy number are enriched among functions critical for adaptive evolution in *Arabidopsis halleri*

**DOI:** 10.1186/s12864-016-3319-5

**Published:** 2016-12-22

**Authors:** Vasantika Suryawanshi, Ina N. Talke, Michael Weber, Roland Eils, Benedikt Brors, Stephan Clemens, Ute Krämer

**Affiliations:** 1 Department of Plant Physiology, Ruhr University Bochum, Universitätsstrasse 150, Bochum, 44801 Germany; 2 Max Planck Institute of Molecular Plant Physiology, Am Mühlenberg 1, Potsdam, 14476 Germany; 3 Department of Plant Physiology, University of Bayreuth, Universitätsstrasse 30, Bayreuth, 95447 Germany; 4 Division of Theoretical Bioinformatics, DKFZ, Im Neuenheimer Feld 280, Heidelberg, 69121 Germany; 5 BioQuant, University of Heidelberg, Im Neuenheimer Feld 267, Heidelberg, 69120 Germany; 6 Institute of Pharmacy and Molecular Biotechnology, University of Heidelberg, Im Neuenheimer Feld 364, Heidelberg, 69120 Germany

**Keywords:** Cross-species, Array-CGH, Metal hyperaccumulation, CNV, *Arabidopsis halleri*, Toll-Interleukin Receptor-Nucleotide Binding Site-Leucine Rich Repeat (TIR-NBS-LRR) protein family, *Resistance* genes (*R* genes)

## Abstract

**Background:**

Gene copy number divergence between species is a form of genetic polymorphism that contributes significantly to both genome size and phenotypic variation. In plants, copy number expansions of single genes were implicated in cultivar- or species-specific tolerance of high levels of soil boron, aluminium or calamine-type heavy metals, respectively. *Arabidopsis halleri* is a zinc- and cadmium-hyperaccumulating extremophile species capable of growing on heavy-metal contaminated, toxic soils. In contrast, its non-accumulating sister species *A. lyrata* and the closely related reference model species *A. thaliana* exhibit merely basal metal tolerance.

**Results:**

For a genome-wide assessment of the role of copy number divergence (CND) in lineage-specific environmental adaptation, we conducted cross-species array comparative genome hybridizations of three plant species and developed a global signal scaling procedure to adjust for sequence divergence. In *A. halleri*, transition metal homeostasis functions are enriched twofold among the genes detected as copy number expanded. Moreover, biotic stress functions including mostly disease *Resistance* (*R*) gene-related genes are enriched twofold among genes detected as copy number reduced, when compared to the abundance of these functions among all genes.

**Conclusions:**

Our results provide genome-wide support for a link between evolutionary adaptation and CND in *A. halleri* as shown previously for *Heavy metal ATPase4*. Moreover our results support the hypothesis that elemental defences, which result from the hyperaccumulation of toxic metals, allow the reduction of classical defences against biotic stress as a trade-off.

**Electronic supplementary material:**

The online version of this article (doi:10.1186/s12864-016-3319-5) contains supplementary material, which is available to authorized users.

## Background

Genetic and epigenetic variation form the basis for local adaptation and speciation processes, and are becoming increasingly accessible through advances in genomic and bioinformatic tools. The advent of microarray and ultra-high throughput sequencing (UHTS) technologies have thus brought about a renewed interest in evolutionary questions, with a prospect for gaining novel insights at the whole-genome level. These opportunities have spurred genome-wide surveys of single nucleotide polymorphisms (SNPs) [1] and methylation polymorphisms in many organisms including plants, for example in multiple accessions of the genetic model organism *Arabidopsis thaliana* and in closely related species [2–6]. In attempts to identify causative genetic changes in plant adaptations, classical linkage analysis and genome-wide association studies (GWAS) have successfully mapped traits governing the performance under local environmental conditions to SNPs at specific loci [7, 8]. Structural variation in the form of gene copy number variation (CNV) polymorphism is an influential component of natural genetic diversity that markedly contributes to phenotypic variation [9]. However, CNV has been addressed in noticeably fewer studies because of technical difficulties in its comprehensive and reliable assessment [10, 11].

Short CNVs consisting of insertions or deletions below 1 kb in size can be readily detected based on UHTS technologies. However, the identification of CNVs comprising from 1 kb up to one or multiple genes has generally remained challenging. Genome-wide analyses in human and other mammalian model organisms revealed CNVs to be much more abundant than previously known, e.g. affecting 10% of the mouse genome and 12% of the human genome (reviewed in [12]). CNVs have been implicated in human disease etiology, and evidence for adaptive CNVs is also emerging [13]. In comparison to mammalian genomes, gene duplications and deletions especially from whole genome duplications appear to be even more abundant in plant genomes [14]. Single-gene and segmental duplications as well as whole-genome duplications have been hypothesized to propel adaptive evolution and speciation. In plants, this view is supported by recent reports on cultivar-specific boron tolerance in barley [15], aluminium tolerance in maize [16] and species-wide heavy metal tolerance in the wild plant *Arabidopsis halleri* [17, 18], all supporting the role of gene copy number expansion in plant adaptation to abiotic stress. Population genomic data, for example from *Arabidopsis thaliana* and *Zea mays*, have identified an unexpectedly high abundance of CNVs [11, 19], generating interest in the contribution of structural mutations to genome plasticity. Ten percent of maize genes were found to exhibit copy number polymorphisms, and an experimental evolution study in *A. thaliana* reported de novo structural mutations resulting in 400 copy number variant genes after only 5 generations [20]. Although between-species genome comparisons have remained difficult to date, the few existing studies have supported the hypothesis that gene copy number expansions, and especially those involving tandem duplications [21], might underlie plant adaptations to environmental stress [22]. Given that novel functions are much more likely to be generated by adaptive specialization of one of several pre-existing copies of a duplicated gene than by an entirely novel gene [23, 24], such comparative studies are key to understanding the patterns of genomic polymorphisms associated with adaptation and speciation.

**Fig. 1:**
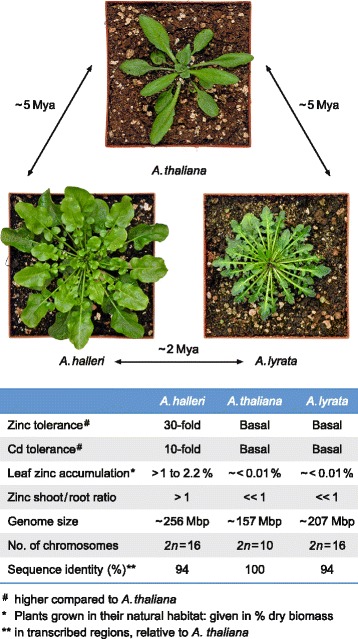
Comparison of the metal hyperaccumulator species *Arabidopsis halleri* to the closely related non-hyperaccumulator species *A. lyrata* and *A. thaliana*. A representative photograph is shown for each species, together with the estimated evolutionary distances separating them, given as the divergence times from a common ancestor [96]. Listed below are key phenotypic and genomic characteristics. Mya, million years ago

The availability of a well-annotated genome sequence and a wealth of knowledge on gene functions for *Arabidopsis thaliana*, as well as for several closely related species that have diverged over short evolutionary timespans, render Arabidopsis a suitable model genus to study adaptation and speciation processes [25, 26]. One of its species is *Arabidopsis halleri* — a wild outcrossing, Zn and Cd hyperaccumulating and hypertolerant species that is naturally found on both highly metal-contaminated and non-contaminated soils (Fig. 1) [27]. Its genome is expected to be about 25% larger than that of its non-accumulating, non-tolerant sister species *A. lyrata*, and about 65% larger than the size of the genome of *A. thaliana*, from which both species diverged between 5.8 [28] and 13 million years ago [29]. The striking phenotypic contrast between *A. halleri* and the two other closely related species, despite a high sequence similarity within coding regions [30], provides an exceptional opportunity to elucidate molecular evolutionary patterns reflecting the influence of natural soil characteristics on adaptation and speciation.

Previous cross-species transcriptomics studies identified a number of differentially expressed candidate genes for the metal hyperaccumulation/hypertolerance trait of *A. halleri* [30–32]. Among these, *Heavy Metal ATPase 4* (*HMA4*), which encodes a metal pump that acts as an exporter of Zn^2+^ and Cd^2+^ from specific cell types, was shown to be necessary both for the hyperaccumulation of Zn and for the full extent of Zn and Cd hypertolerance [18]. Strongly increased *HMA4* transcript levels in *A. halleri* were attributed to a lineage-specific tandem triplication combined with *cis*-regulatory mutations [18]. An analysis of sequence polymorphism in the genomic region of *HMA4* gene copy number expansion demonstrated strong positive selection, as well as selection for enhanced *HMA4* gene product dosage [17]. Another candidate gene, *Nicotianamine Synthase 2* (*NAS2*), encodes an enzyme that catalyses the biosynthesis of the low-molecular-weight metal chelator nicotianamine from S-adenosyl methionine, and was shown to contribute to Zn hyperaccumulation [33]. In addition to *HMA4*, several other transition metal homeostasis candidate genes of *A. halleri* were demonstrated to be copy number expanded through the DNA gel (Southern) blot technique [31, 34].

The objective of the work presented here was to identify genes exhibiting copy number expansion in *A. halleri* at a genome-wide scale, in relation to the known species-specific extreme traits. We conducted a survey of gene copy number divergence (CND) in *A. halleri* relative to *A. thaliana* by employing array-comparative genomic hybridization (array-CGH) in a cross-species manner using the ATH1 microarray designed for *A. thaliana*. In order to test whether the identified CNDs are species- and thus possibly trait-specific, our analysis included *A. lyrata* as a third species, which is a non-tolerant non-hyperaccumulator like *A. thaliana*, but shares with *A. halleri* an equal phylogenetic distance from *A. thaliana*. We devised a novel routine for evaluating cross-species array-CGH data, which is based on the quantification and subsequent global correction of the effects of sequence divergence on hybridization signal intensities. Our procedure operates without loss of probe information, which is crucial for retaining statistical power for CNV estimation further downstream. Our predictions of genic CNDs were validated against a small set of genes with known copy number in *A. halleri* [31] and against a set of genes predicted to be copy number expanded or reduced according to the *A. lyrata* reference genome sequence [35]. Gene copy number expansions in *A. halleri*, but not in *A. lyrata*, were found to be significantly enriched for metal homeostasis functions. Conversely, biotic stress functions were significantly enriched among genes exhibiting copy number reduction in *A. halleri*, but not in *A. lyrata*. These results suggest that between-species divergence in gene copy numbers reflects adaptive evolution of metal hyperaccumulation, a species-specific trait of *A. halleri* that has been proposed to provide an elemental defence against biotic stress [36, 37].

## Results

Metal hyperaccumulation and hypertolerance in *A. halleri* have previously been attributed to the constitutively enhanced expression of a number of metal homeostasis genes, several of which were additionally shown to be expanded in genomic copy number through DNA gel blots [31], BAC sequencing [18, 38] or other methods [34]. Here, the technique of cross-species array-CGH was employed for a genome-wide assessment of between-species divergence in gene copy number. Fragmented and labelled nuclear genomic DNA samples from *A. thaliana* and from the two closely related heterologous species *A. halleri* and *A. lyrata*, were hybridized in duplicate to Affymetrix ATH1 GeneChip^®;^ microarrays (see Fig. 1) [39].

**Fig. 2:**
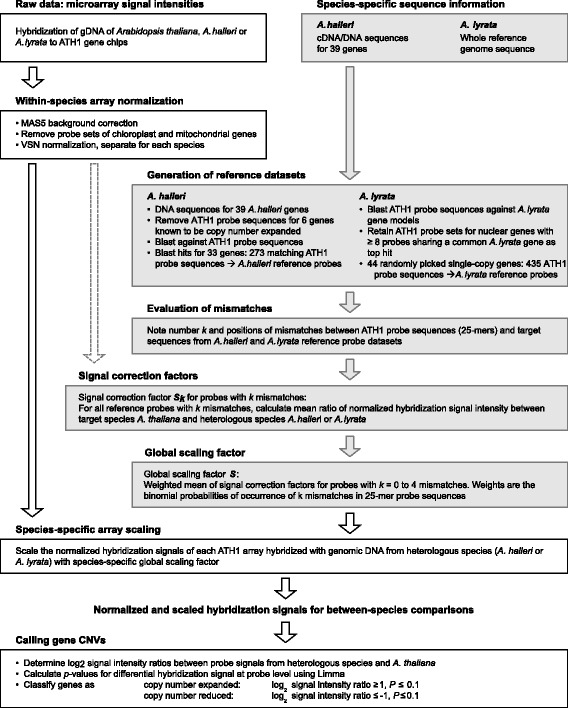
Overview of the data analysis workflow. Flowchart summarizing our two-step normalization approach for the processing of cross-species genomic hybridization data, consisting of within-species normalization and global scaling of signals through species-specific signal correction factors, followed by the final prediction of copy number divergent genes. Grey arrows and backgrounds mark the auxiliary steps taken for the determination of species-specific global scaling factors with the aid of reference gene datasets

The challenge in cross-species hybridizations of genomic DNA is that the target gene sequences of the heterologous species inevitably contain mismatches relative to the probe sequences of the reference species on the microarray, thus reducing the efficiency of hybridization and resulting in lowered signals [40, 41]. We employed a novel approach to correct this bias by using a signal adjustment strategy, which - unlike previous methods [42, 43] — accounts for sequence mismatches through a global adjustment of cross-species hybridization signal intensities. In brief, we implemented a two-step normalization scheme (Fig. 2). The first step was a conventional within-species normalization, which was applied to raw signal intensities from each pair of two replicate microarray hybridizations of the same target species. The second step was an adjustment of normalized signal intensities through the calculation and application of a species-specific global scaling factor for compensating the effects of sequence divergence from *A. thaliana*.

To implement our strategy, we began by establishing a representative subset of probes for which curated sequence data was available from *A. halleri*, termed reference dataset (see Fig. 2; Additional file 1). A similar reference dataset was also generated at random from the available reference genome sequence of *A. lyrata*. For each heterologous target species, the signal correction factor was calculated from the statistical distribution of the occurrence of mismatches and the ensuing effect on hybridization signal intensity as measured in the respective reference dataset. Subsequently, the normalized and corrected cross-species hybridization data were analysed for differential signals between species in order to identify putative copy number divergent genes. Finally, a comparison between copy number alterations in *A. lyrata* and *A. halleri* enabled us to identify species-specific copy number alterations.

### Consequences of inter-species sequence divergence for mismatch occurrence between probe sequences and heterologous target sequences

**Fig. 3:**
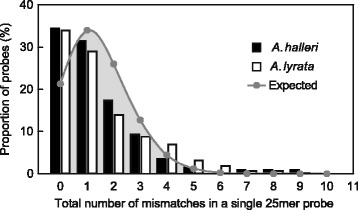
Frequency distribution of mismatch occurrence between microarray probe sequences and heterologous target gene sequences. Shown is the percentage of *A. thaliana* probes on the ATH1 array that display no mismatches up to 11 mismatches (observed maximum) when hybridized to non-*A. thaliana* genomic DNA from either *A. halleri* (*black bars*) or *A. lyrata* (white bars). The expected frequency distribution (binomial) is shown by the grey line, and was calculated based on the average coding sequence identity (94%) within transcribed regions between *A. halleri* and *A. thaliana*

For the adjustment of microarray signals in cross-species array-CGH, we generated one reference dataset of representative, curated sequence data for each of the two heterologous target species. The *A. halleri* reference dataset comprised 33 genes, yielding 273 matching probe sequences on the microarray (Fig. 2, Additional file 2, Additional file 1; see Methods). Because of the lack of a reference genome, these data corresponded to previously obtained sequences from *A. halleri* ssp. *halleri* (Langelsheim/Germany) [30, 31, 33]. The *A. lyrata* reference dataset comprised 44 genes with 435 matching probe sequences on the microarray, obtained from the published reference genome [35] (Fig. 2, Additional file 2, Additional file 1; see Methods). The number and positions of mismatches between each heterologous target sequence and the corresponding microarray probe sequence was determined (see Additional file 1). For *A. halleri* and *A. lyrata*, respectively, 34 and 35% of all probe sequences were fully conserved across species, 33 and 29% contained only a single mismatch with respect to the 25 nucleotide-long probe sequence, and 29 and 30% of sequences contained between 2 and 4 mismatches compared to the corresponding probe sequence (Fig. 3, Additional file 2).

We computed the expected distribution of the total number of mismatches of a given nucleotide sequence with respect to the probe sequence on the microarray (See Methods). The expected distribution (binomial) of mismatches in cross-species hybridization of *A. halleri* gDNA closely matched our observations for the reference dataset (Pearson’s *χ* ^2^=0.985, df = 4, *P* = 0.37; Fig. 3). Both the observed and expected probabilities of the occurrence of more than 4 mismatches by comparison to the probe sequence were negligible (3 and 6% for *A. halleri* and *A. lyrata* reference dataset, respectively, and 1.5% expected). Finally, the observed mismatch distributions for *A. halleri* and *A. lyrata* were highly similar to each other (Pearson’s *χ* ^2^= 0.979, df = 4, *P* = 0.44), thus confirming similar levels of sequence divergence from *A. thaliana* (see Fig. 1) and allowing us to use the expected distribution of mismatches calculated for *A. halleri* also in *A. lyrata* hybridizations.

Both the *A. halleri* and *A. lyrata* lineages are thought to have diverged from the common ancestor with *A. thaliana* at the same point in the past (see Fig. 1). Our observations support the theory that a correlation exists between the levels of sequence divergence and actual phylogenetic distances between species, as was estimated, for example, based on cross-species array-CGH data [44].

### Quantification of effects of sequence mismatches on signal intensities in cross-species microarray hybridization

**Fig. 4:**
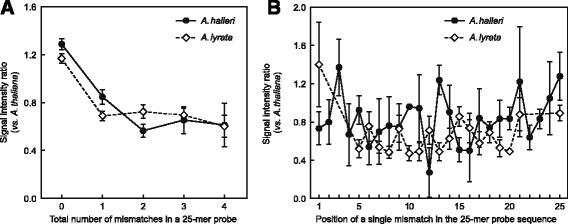
Dependence of hybridization signal intensity on number and position of mismatches with respect to the probe sequence on the ATH1 array. **a** Values are arithmetic means (± SD; *n* = 8 to 94) of background-corrected raw probe signal intensity ratios for non-*A. thaliana* gDNA relative to *A. thaliana* gDNA hybridizations, shown as a function of the total number of mismatches of the heterologous target sequence compared to the corresponding *A. thaliana* 25-mer probe sequence. **b** Independence of hybridization signal intensity from the position of a single mismatch with respect to the probe sequence. Values are arithmetic means (± SD; *n* = 2 to 6) of background-corrected raw probe signal intensity ratios for non-*A. thaliana* gDNA relative to *A. thaliana* gDNA hybridizations, shown as a function of mismatch position in the heterologous target sequence compared to the corresponding *A. thaliana* probe sequence. Black circles represent the representative *A. halleri* reference dataset; white diamonds represent the representative *A. lyrata* reference dataset

Sequence mismatches are known to be the single most confounding factor biasing the signals of cross-species array hybridizations. A previous study has estimated sequence mismatches to account for at least 40% of the average noise in microarray hybridization [45], and several studies confirm mismatches as the primary cause of failure of conventional normalization techniques in cross-species microarray data analysis [40, 46]. As a result of sequence divergence, sequence mismatches are expected to reduce the hybridization efficiency of genomic DNA from *A. halleri* and *A. lyrata* to the *A. thaliana* probe sequences on the ATH1 microarray, resulting in lowered overall hybridization signal intensity. After background correction of raw data (see Methods), we examined the influence of the total number and positions of mismatches on the normalized hybridization signal intensities using the probe signal intensities from our *A. halleri* and *A. lyrata* reference datasets. As expected, hybridization signal intensity decreased with increasing number of mismatches in a probe. The largest decrease in signal intensity by 34 and 40% in *A. halleri* and *A. lyrata*, respectively, was observed for a single mismatch (Fig. 4 a). Additional mismatches had only small effects, with a total of four mismatches resulting in a further reduction of probe signal intensity by 20 and 7% in *A. halleri* and *A. lyrata*, respectively. There were only minor differences between *A. halleri* and *A. lyrata* in the dependence of signal intensity on the number of mismatches. Note that the published *A. lyrata* reference genome, which was used here to determine the number of mismatches between *A. lyrata* reference dataset target sequences and the corresponding ATH1 probe sequences, was from the North American subspecies *lyrata*, whereas our hybridization experiments were conducted with the European *ssp. petrea* [35]. A stark reduction in diversity has been reported in *A. lyrata ssp. lyrata* by comparison to *A. lyrata ssp. petrea*, and several studies (reviewed in [47]) report differentiation between the two sub-species that have been isolated from each other for between 35 and 47 thousand years [47].

Surprisingly, we observed a noisy profile of signal intensity over different positions of a single mismatch along the probe sequence instead (Fig. 4 b). The expected sharp drop in signal intensity when a single mismatch is positioned in the centre (13th nucleotide) of a probe sequence, as proposed by Affymetrix for so-called mismatch (MM) probes [39], was not detected here. This finding is in agreement with a number of previous studies [48, 49], which have pointed out that experimental data do not conform to this postulate and that, in fact, for some probes signal intensity was even found to be higher for MM probes than for perfectly matching (PM) probes [49]. Consequently, position-based effects on hybridization signal intensity are hard to construct, and accordingly, the most popular normalization methods no longer take the information from MM probes into account. Therefore, for our between-species normalization strategy, we did not consider the influence of sequence mismatch position. We estimated the incremental signal correction factor *S*_*k*_ for a probe with *k* mismatches as the average of the ratio of normalized hybridization signal intensity of *A. thaliana* to the respective signal intensity of the heterologous species. For each probe containing 0 to 4 mismatches, incremental signal correction factors were weighted by their probability of occurrence (see Fig. 3), followed by the calculation of the arithmetic mean to yield species-specific global scaling factors (see Fig. 2). These global signal correction factors of 1.22 for hybridizations of *A. halleri* gDNA and 1.13 for hybridizations of *A. lyrata* gDNA were employed to scale the hybridization signal intensities of the respective cross-species microarray hybridizations.

### Cross-species normalization and validation of copy number divergent genes

**Fig. 5:**
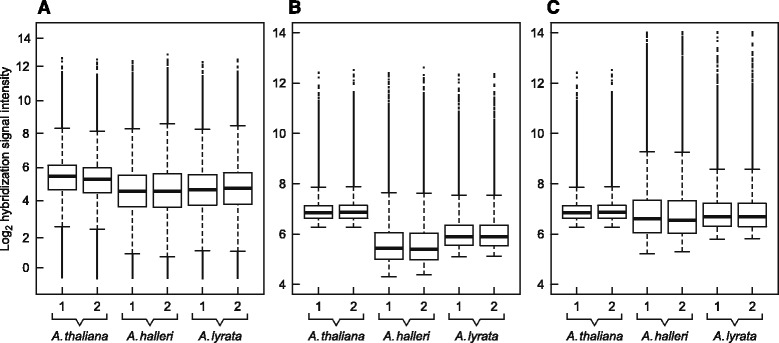
Distribution of signal intensities before and after normalization and scaling. Boxplot of (**a**) background-corrected raw hybridization signal intensities, (**b**) normalized signal intensities after VSN normalization of the replicate arrays of each species, respectively, and (**c**) signal intensities after the application of species-specific global scaling factors to the normalized data. Boxes show median, and upper and lower quartiles, of *L* *o* *g*_2_ probe signal intensities for each gDNA hybridization. Upper and lower horizontal bars mark all values lying within 1.5 times the inter-quartile range. Replicate hybridizations are denoted 1 and 2 and grouped by species

The median raw signal intensities for the heterologous species *A. halleri* and *A. lyrata* were lower than those for the ATH1 target model species *A. thaliana*, namely by 42 and 36%, respectively (Fig. 5 a). After applying conventional within-species VSN normalizations, median normalized signal intensities were more uniform across replicates within each species (Fig. 5 b). However, the differences between species were large, with median signal intensities for *A. halleri* and *A. lyrata* which were 63 and 49% lower, respectively, than for *A. thaliana*. Upon the subsequent between-species scaling of VSN-normalized signal intensities from the *A. halleri* and *A. lyrata* hybridizations employing species-specific global scaling factors (see Methods), median signal intensities became more similar across species and remained 17% lower for *A. halleri* and 11% lower for *A. lyrata*, respectively, than for *A. thaliana* (Fig. 5 c). Following normalization and scaling of hybridization signals, 1,195 and 217 copy number expanded genes (CNEs) were identified in *A. halleri* and *A. lyrata* (Additional file 3), respectively (*L* *o* *g*_2_ ratio ≥ 1 scaled hybridization signal intensities of *A. halleri* or *A. lyrata* relative to the reference species *A. thaliana*; adjusted *P* ≤ 0.1). Furthermore, 946 genes predicted to be copy number reduced (CNRs) were identified in *A. halleri* and 479 in *A. lyrata* (*L* *o* *g*_2_ ratio ≤ -1 scaled hybridization signal intensities of *A. halleri* or *A. lyrata* relative to the reference species *A. thaliana*; *P* ≤ 0.1). Overall, 145 CNEs and 177 CNRs are shared between *A. halleri* and *A. lyrata* compared to the reference species *A. thaliana* (see also Additional file 3). Thus, based on *A.* *thaliana* as a reference, an about 3-fold larger number of copy number divergent genes was detected in *A. halleri* than in *A. lyrata*, whereas nucleotide sequence divergence from *A. thaliana* was similar in both heterologous species within transcribed regions (see Figs. 1 and 3). The observed difference between *A. halleri* and *A. lyrata* was not merely a spurious result caused by a higher level of polymorphism between the two replicate *A. halleri* samples than between the *A. lyrata* replicates. This was confirmed by performing all data processing steps with two additional pairs of *A. halleri* replicates, each consisting of either one of the two single *A. halleri* hybridizations and one *A. halleri* replicate generated *in silico* by simulating between-replicate variation as observed in *A. lyrata*, respectively (Additional file 4). Consequently, our results suggest that the rate of either acquisition or maintenance of gene copy number changes in the genome can differ between closely-related lineages or species. This is in stark contrast to the general stability of base substitution rates normalized to genome size and generation [50].

To evaluate the reliability of our predicted CNDs, we compared our results to genes of known copy number status. For *A. halleri*, we used a set of 14 genes (see Additional file 5, Methods). The evaluation of microarray-based predictions of cross-species gene CND against known copy number status (Additional file 5) indicated 87.5% specificity, 85.7% precision and 66.7% sensitivity of our cross-species array-CGH based CND estimation. For *A. lyrata*, a complete reference genome sequence is available [35]. This provides an opportunity for more extensive data validation by comparing our predictions of gene CNDs with predictions based on the reference genome sequence. Orthology predictions retrieved from Ensembl Plants indicated that, relative to all *A. thaliana* genes represented on the ATH1 GeneChip, 1,335 orthologous genes of *A. lyrata* are copy number expanded, whereas 4,037 genes are reduced in copy number or deleted (Additional file 6). From this set of genes, we further chose the subset of highly conserved multi-copy genes of ≥ 95% average sequence identity between transcribed regions of all paralogs with respect to their *A. thaliana* ortholog. Our final reference dataset of highly conserved copy number expanded genes in *A. lyrata*, as predicted based on Ensembl Plants, contained 117 genes. By comparison, our method yielded 99.5% specificity, 5.3% precision and 10.3% sensitivity (Table 1). These scores suggest that our procedure is conservative, making predictions with high reliability, and sacrificing sensitivity for a higher specificity. A direct comparative assessment of the performance of our array-CGH based method for *A. halleri* and *A. lyrata* is not possible because of the differing qualities of experimental evidence underlying the two datasets available for validation.

**Table 1:** Validation of array-CGH results against highly conserved^a^ genes predicted to be copy number expanded (CNEs) in *A. lyrata*

Array-CGH prediction method	Total number of CNEs detected	No. of CNEs detected (117 ; 98)^b^		Sensitivity (% positives detected out of predicted positives)		Specificity (% negatives detected out of predicted negatives)		Precision (% true positives out of total no. detected)^c^	
		E	E-A	E	E-A	E	E-A	E	E-A
Present manuscript	217	12	12	10.3	12.2	99.5	99.6	5.3	5.3
Darby et al. 2011^d^	949	10	8	8.5	8.2	99.5	99.6	1.3	1.1
Machado et al. 2010^e^	298	7	5	5.9	5.1	99.5	99.6	2.9	2.1

^a^ *A. lyrata* genes sharing ≥ 95% sequence identity with their closest *A. thaliana* homologue [68] are termed highly conserved (compare Additional file 7)

^b^Headers of half-columns refer to total number of CNEs predicted by Ensembl Plants (E; Vilella et al. 2009) alone or additionally by *A. lyrata* genome analysis (E-A; Hu et al. 2011), respectively, as given in parentheses here. Shown are commonalities with these two groups of genes (same column, below) or data referring to these two groups of genes (columns to the right)

^c^True positive is a CNE detected based on array-CGH that was previously predicted to be a CNE by Ensembl Plants [68] alone, or additionally by *A. lyrata* genome [35] analysis

^d^[42]

^e^[43]

We compared the performance of our array-CGH based approach with that of the two previous studies that also aimed at estimating CND using the array-CGH technique [42, 43]. We reproduced the normalization and scaling strategies of Machado and Renn (2010) and Darby et al. (2011) as described [42, 43], with few small modifications necessary to apply these methods to our array-CGH platform (see Methods). The method of Darby et al. (2011) resulted in the prediction of a 2.47-fold elevated number of gene copy number expansions. Out of the two previously published methods, maximum sensitivity, specificity and precision of the detection of copy number expansion among highly conserved genes were 8.5, 99.5 and 2.1%, respectively, all inferior to our method (10.3%, 99.5%, 5.3%, Table 1). Even for the genes that are not highly conserved but predicted to be copy number expanded concordantly by both Ensembl Plants and the *A. lyrata* genome project, our method reports higher sensitivity, specificity and precision – 5, 99.1 and 8.8% respectively than previous studies [42, 43] – 3.9, 98.7 and 3.7% (Additional file 7A). Specificity and precision of our method were also superior concerning copy number reductions or gene deletions (Additional file 7B).

### Functional analysis of copy number divergent genes of *A. halleri*

**Fig. 6:**
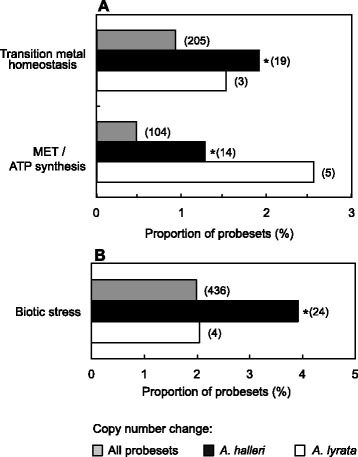
Significantly enriched functional classes among (**a**) copy number expanded and (**b**) copy number reduced genes of *A. halleri*. *Bars/brackets* represent the proportion/number of genes in each functional class among all copy number expanded or reduced genes of *A. halleri* (*black*) or *A. lyrata* (*white*), and among all nuclear genes represented on the array (*grey*) for comparison. *Asterisks mark* statistically significant enrichment by comparison to the set of nuclear genes represented on the ATH1 microarray (Fischer’s exact test, *P* ≤ 0.05, Benjamini-Hochberg correction for multiple testing). The analysis was based on MapMan [51] functional categories (see Additional file 8). Shown are only top-level categories of the ontological hierarchy (see text)

After identifying the sets of genes exhibiting copy number divergence by comparison to the reference species *A. thaliana* in either of the two heterologous species according to array-CGH, we evaluated these for any enrichment of functional categories using the MapMan ontology [51]. Copy number expanded genes of *A. halleri* showed a statistically significant enrichment for transition metal homeostasis-related gene functions (1.92%, *P* ≤ 0.05; Table 2A; see Additional file 8), and for mitochondrial electron transport/ATP synthesis-related functions (1.28%, *P* ≤ 0.05; Table 2B), relative to all genes represented on the array (0.94 and 0.47%, respectively; Fig. 6). Among copy number reduced genes of *A. halleri*, there was a significant enrichment for biotic stress-related functions (3.92% by comparison to 1.99% on the entire array, *P* ≤ 0.05; Table 2C). In contrast, none of these functional categories was detected to be significantly enriched among genes divergent in copy number in *A. lyrata* by comparison to *A. thaliana*. In the light of the two extreme traits specific to *A. halleri* — namely Zn/Cd hyperaccumulation and associated hypertolerance — the genome-wide overrepresentation of metal homeostasis genes among copy number expanded genes is remarkable (see Fig. 6, Table 2A). Indeed, a high occurrence of copy number expansion was reported among *Arabidopsis halleri* metal homeostasis candidate genes identified through the presence of elevated transcript levels in *A. halleri* compared to *A. thaliana* [31]. Moreover, copy number expansion is known to contribute to high *HMA4* transcript levels in *A. halleri*, which in turn are necessary for both metal hyperaccumulation and the full extent of metal hypertolerance [18]. *HMA4* gene copy number expansion is not limited to *A. halleri*, but also found in the Zn/Cd hyperaccumulator species *Noccaea caerulescens*, similarly associated with strongly elevated transcript levels [52]. *A. halleri MTP1* is another copy number-expanded candidate gene, for which several lines of evidence suggest an involvement in Zn hypertolerance [32, 34, 38, 53]. It was not known to date whether these findings on individual candidate genes pertain at the genome-wide level, but this is now supported by array-CGH data presented here. Our data additionally confirm the previous finding of *ZIP6* copy number expansion [31]. In contrast, our array-CGH analysis did not detect *HMA4* as copy number expanded in *A. halleri*, although this is well established. One of the transition metal homeostasis candidate genes newly identified to be copy number expanded in *A. halleri* is *NAS2*, which was demonstrated to be highly expressed in roots of *A. halleri* [30] and to contribute to Zn hyperaccumulation [33]. Array-CGH also predicts *AhHMA3* to be copy number expanded. This candidate gene was reported as highly expressed in *A. halleri*, and an *AhHMA3* cDNA confers Zn and Cd tolerance upon heterologous expression in yeast [32]. Finally, *AtPCR1* and *AtPCR2* have been implicated in the export of Zn and Cd, respectively, from cells [54, 55] and appear to be copy number expanded in *A. halleri*. Indeed, transcript levels were found to be higher in *A. halleri* than in *A. thaliana* in a previous microarray hybridization study [31], but this was not explicitly reported because of an ambiguous assignment of the probe set to this pair of highly similar *A. thaliana* genes. *SAMS2* encodes an enzyme that catalyses the biosynthesis of the substrate for nicotianamine synthase and was previously identified to be more highly expressed in *A. halleri* than in *A. thaliana*; and it is now identified as copy number expanded. Expanding from these findings, the enrichment of mitochondrial electron transport functions among the genes copy number expanded in *A. halleri* was surprising. A majority of 8 copy number expansions, half of which are shared with *A. lyrata*, affect complex I acting as NADH ubiquinone oxidoreductase in oxidative phosphorylation (Table 2B). A more parsimonious explanation for the common expansions could be that these genes have been deleted in *A. thaliana* relative to the common ancestor. The two genes privately copy number expanded in *A. halleri* have predicted roles in ATP synthesis and cytochrome c biogenesis, respectively, and one is annotated as a mitochondrial dicarboxylate carrier.

**Table 2:** Genes identified to be altered in copy number in *A. halleri* through cross-species hybridization of gDNA onto *A. thaliana* microarrays

Affymetrix probeset ID	AGI locus ID	Short gene name^a^	Gene description	*A. halleri vs. A. thaliana*		*A. lyrata vs. A. thaliana*	
				*L* *o* *g*_2_ FC^b^	*P*-value	*L* *o* *g*_2_ FC^b^	*P*-value
(A)							
Copy number expanded in *A. halleri vs. A. thaliana*(*L* *o* *g*_2_ *F* *C*> 1)							
256055_at	At1g07030	*Mfm1*	Mitoferrin-related, mitochondrial solute carrier (MSC) family	1.23	0.01	-0.05	0.43
262832_s_at	At1g14870; At1g14880	*PCR1; PCR2*	Plant cadmium resistance 1/2	1.69	0.00	1.08	0.00
247824_at	At5g58460	*CHX25*	Member of Na^+^/H^+^ antiporter family	2.50	0.00	0.24	0.63
261448_at^c^	At1g21140		Vacuolar iron transporter 1 (VIT1)-related	1.27	0.00	0.76	0.00
262958_at	At1g54410	*ITP2*	Ricinus iron transport protein 2-related	1.21	0.08	1.3	0.00
262936_at	At1g79400	*CHX2*	Cation/H^+^ exchanger 2	1.13	0.03	0.53	0.16
267304_at^d^	At2g30080	*ZIP6*	ZRT-, IRT-like protein 6	1.12	0.08	0.46	0.55
263831_at	At2g40300	*FER4*	Ferritin 4	1.22	0.00	0.5	0.37
266718_at^c,d^	At2g46800	*MTP1*	Metal transport/tolerance protein 1	2.5	0.00	0.24	0.63
259008_at	At3g09390	*MT2a*	Metallothionein 2a	1.22	0.00	0.57	0.00
252697_at	At3g43660		Vacuolar iron transporter 1 (VIT1)-related	1.16	0.08	0.78	0.02
251735_at	At3g56090	*FER3*	Ferritin 3	1.3	0.00	0.2	0.63
251733_at	At3g56240	*CCH*	Copper chaperone	1.12	0.00	0.44	0.19
255552_atd	At4g01850	*SAMS2*	S-adenosylmethionine synthetase 2	1.38	0.02	0.51	0.42
254604_at	At4g19070	*AS8*	Cadmium-induced protein AS8	1.13	0.00	0.49	0.05
253658_at^c,d^	At4g30120	*HMA3*	Heavy metal ATPase 3	2	0.00	0.63	0.01
252864_at	At4g39740	*HCC2*	Homologue of yeast copper chaperone Sco1xx	1.06	0.01	0.34	0.66
250991_at	At5g02380	*MT2b*	Metallothionein 2b	1.32	0.05	0.43	0.34
248048_at^c^	At5g56080	*NAS2*	Nicotianamine synthase 2	1.26	0.00	1.07	0.00
Putative copy number expanded in *A. halleri vs. A. thaliana*(*L* *o* *g*_2_ ≤ 1 and > 0.6)							
249334_at	At5g41000	*YSL4*	Yellow stripe like transporter 4	0.99	0.43	0.00	0.08
266336_at	At2g32270	*ZIP3*	ZRT-, IRT-like^$^ protein 3	0.97	0.28	0.09	0.58
260283_at	At1g80480	*PTAC17*	Plastid transcriptionally active 17, putative Zn metallochaperone	0.96	0.46	0.00	0.05
260551_at	At2g43510	*TI1*	Trypsin inhibitor 1, defensin-like protein family	0.95	0.24	0.00	0.74
250944_at^d^	At5g03380	*HIPP6*	Putative metallochaperone-like protein	0.95	0.67	0.00	0.00
258745_at	At3g05920	*HIPP43*	Putative metallochaperone-like protein	0.93	0.45	0.00	0.06
253964_at	At4g26480	*NAS-like*	Protein with NAS domain and KH domain	0.93	0.37	0.01	0.14
247331_at	At5g63530	*FP3*	Farnesylated protein 3, metal-binding	0.92	0.45	0.00	0.09
266091_at	At2g37920	*COPT4, EMB1513*	Copper transporter 4, embryo defective 1513	0.92	0.68	0.01	0.01
259871_at^c^	At1g76800		Vacuolar iron transporter 1 (VIT1)-related	0.91	0.18	0.02	0.85
256930_at^d^	At3g22460	*OASA2*	O-acetylserine thiol lyase isoform A2	0.90	0.42	0.06	0.14
253413_at^c^	At4g33020	*ZIP9*	ZRT-, IRT-like^$^ protein 9	0.89	0.07	0.03	0.79
266115_at	At2g02140	*PDF2.6*	Plant defensin 2.6	0.86	0.46	0.00	0.01
258415_at	At3g17390	SAMS3/ MAT4*	S-adenosylmethionine synthetase 3	0.86	0.09	0.00	0.95
260489_at	At1g51610	*MTP7*	Metal transport protein 7	0.86	0.75	0.00	0.00
260913_at^d^	At1g02500	*SAMS1*	S-adenosylmethionine synthetase 3	0.85	0.44	0.00	0.03
262324_at	At1g64170	*CHX16*	Cation/H^+^ exchanger, CPA2 family	0.84	0.38	0.07	0.36
257365_x_at^c,d^	At2g26020	*PDF1.2b*	Plant defensin 1.2b	0.83	0.05	0.02	0.69
249255_at^c^	At5g41610	*CHX18*	Cation/H^+^ exchanger, CPA2 family	0.77	0.63	0.00	0.01
263838_at^c,d^	At2g36880	MAT3**	Methionine adenosyltransferase 3	0.74	0.45	0.00	0.01
247128_at	At5g66110	*HIPP27*	Putative metallochaperone-like protein	0.73	0.35	0.00	0.02
264644_at	At1g08960	*CAX11*	Ca2^+^/H^+^ exchanger 11	0.67	0.60	0.00	0.00
252694_at^c^	At3g43630		Vacuolar iron transporter 1 (VIT1)-related	0.67	0.60	0.02	0.00
261135_at	At1g19610	*PDF1.4*	Plant defensin 1.4	0.67	0.42	0.00	0.01
260601_at	At1g55910	*ZIP11*	ZRT-, IRT-like^$^ protein 11	0.65	0.02	0.02	0.57
257054_at	At3g15353	*MT3*	Metallothionein 3	0.62	0.30	0.01	0.12
258987_at^d^	At3g08950	*HCC1*	Homologue of yeast copper chaperone Sco1^*∞*^	0.61	0.23	0.00	0.25
(B)							
Affymetrix probeset ID	AGI locus ID	Short gene name	Gene description	*A. halleri vs. A. thaliana*		*A. lyrata vs. A. thaliana*	
				*L* *o* *g*_2_ FC^b^	*P*-value	*L* *o* *g*_2_ FC^b^	*P*-value
264097_s_at	At1g16700; At1g79010	—; *TYKY*	Complex I & 23 kDa subunit; *α*-helical ferredoxin	2.18	0.00	1.11	0.00
261489_at	At1g14450	*B12-1*	Complex I & B12 subunit	1.84	0.00	1.19	0.00
254120_at	At4g24570	*DIC2*	Dicarboxylate carrier 2	1.80	0.00	1.11	0.00
245715_s_at	At5g08670; At5g08690		ATP synthase *β*-subunit	1.78	0.00	0.64	0.01
266512_at	At2g47690		Complex I & 14 kDa subunit; Fe-S subunit 5	1.73	0.00	1.24	0.00
258847_at	At3g03100	*B17.2*	Complex I & 17.2 kDa subunit	1.69	0.06	1.17	0.07
260767_s_at	At1g49140; At3g18410	*PDSW*; —	Complex I & 12 kDa subunit NDUFS6; PDSW subunit	1.34	0.00	0.76	0.00
256679_at	At3g52300	*ATPQ*	ATP synthase D chain	1.26	0.00	0.03	0.66
262397_at	At1g49380		Cytochrome *c* biogenesis protein family	1.21	0.01	0.48	0.21
249627_at^d^	At5g37510	*EMB1467*	Complex I & subunit of the 400 kDa subcomplex; Embryo defective 1467	1.18	0.00	0.76	0.00
246309_at	At3g51790	*CCME*	Orthologue of *E. coli* CcmE heme chaperone in cytochrome *c* maturation	1.10	0.01	0.60	0.06
252864_at	At4g39740	*HCC2*	Homologue of yeast copper chaperone Sco1^*∞*^	1.06	0.01	0.34	0.66
263375_s_at	At2g20530; At4g28510	*PHB6; PHB1*	Complex I & Prohibitin 6; Prohibitin 1	1.02	0.00	0.36	0.04
256267_at	At3g12260	*B14*	Complex I & LYR family of Fe/S cluster biogenesis protein	1.02	0.01	0.31	0.32
(C)							
Affymetrix probeset ID	AGI locus ID	Short gene name^a^	Gene description	*A. halleri vs. A. thaliana*		*A. lyrata vs. A. thaliana*	
				*L* *o* *g*_2_ FC^b^	*P*-value	*L* *o* *g*_2_ FC^b^	*P*-value
245219_at	At1g58807; At1g59124		Disease resistance protein (CC-NBS-LRR^#^ class) family	–1.50	0.00	–1.30	0.00
248851_s_at	At5g46260; At5g46490		Disease resistance protein (TIR-NBS-LRR^#^ class) family	–1.37	0.00	–1.26	0.00
262374_s_at	At1g72910; At1g72930		TIR domain-containing protein	–1.20	0.00	–0.70	0.00
255060_at	At4g09430		TIR-NBS-LRR^#^ class	–1.20	0.00	–0.84	0.00
250069_at	At5g17970		TIR-NBS-LRR^#^ class	–1.20	0.00	–0.70	0.00
250771_at	At5g05400		LRR and NB-ARC^+^ domains-containing disease resistance protein	–1.17	0.00	–0.59	0.00
262362_at	At1g72840		TIR-NBS-LRR^#^ class	–1.12	0.00	–0.76	0.00
245454_at	At4g16920		TIR-NBS-LRR^#^ class	–1.12	0.00	–0.59	0.00
257099_s_at	At3g24982; At3g25020	*RLP40; RLP42*	Receptor like protein 40/42	–1.11	0.00	–0.77	0.00
252648_at	At3g44630		TIR-NBS-LRR^#^ class	–1.11	0.00	–0.59	0.00
250039_at	At5g18370		TIR-NBS-LRR^#^ class	–1.11	0.00	–0.74	0.00
251438_s_at	At3g59930; At5g33355		Defensin-like (DEFL) family	–1.10	0.00	–0.54	0.00
252489_at	At3g46710		NB-ARC^+^ domain-containing disease resistance protein	–1.10	0.00	–0.70	0.00
262363_at	At1g72850		TIR-NBS^#^ class	–1.09	0.00	–0.67	0.00
248973_at	At5g45050	*TTR1; WRKY16*	Tolerant to tobacco ringspot nepovirus	–1.09	0.00	–0.24	0.01
256431_s_at	At3g11010; At5g27060	*RLP34; RLP53*	Receptor like protein 34/53	–1.06	0.00	–0.60	0.00
245674_at	At1g56680		Chitinase family protein	–1.06	0.00	–0.83	0.00
249320_at	At5g40910		TIR-NBS-LRR^#^ class	–1.06	0.00	–0.37	0.00
262364_at	At1g72860		TIR-NBS-LRR^#^ class	–1.05	0.00	–0.64	0.00
255059_at	At4g09420		TIR-NBS-LRR^#^ class	–1.04	0.00	–0.62	0.00
265723_at	At2g32140		TIR domain transmembrane protein	–1.03	0.00	–0.74	0.00
252684_at	At3g44400		TIR-NBS-LRR^#^ class	–1.03	0.00	–0.66	0.00
263572_at	At2g17060		TIR-NBS-LRR^#^ class	–1.02	0.00	0.00	0.33
245457_s_at	At4g16940; At4g16960		TIR-NBS-LRR^#^ class	–1.01	0.00	–0.68	0.00

(A) Transition metal homeostasis. (B) Mitochondrial electron transport/ATP synthesis. (C) biotic stress MapMan functional classes

^a^Short names of genes previously reported to be copy-number expanded in *A. halleri* based on other experimental approaches are given in bold type (see also Additional file 5)

^b^ *L* *o* *g*_2_ fold-change (FC) is underlined for genes that are concordantly copy number expanded/reduced in both *A. halleri* and *A. lyrata*

^c^Higher transcript abundance in roots of *A. halleri vs. A. thaliana* (Supplemental Table 1 of ref. Talke et al. 2006)

^d^Higher transcript abundance in shoots of *A. halleri vs. A. thaliana* (Supplemental Table 2 of Talke et al. 2006)

^*∞*^Synthesis of cytochrome *c* oxidase;

^$^Zinc-regulated transporter, iron-regulated transporter-like; *At3g17390 was annotated as *SAMS4* in ref. Talke et al. 2006; **At2g36880 was annotated as *SAMS3* in Talke et al. 2006; ^&^NADH-ubiquinone oxidoreductase, mitochondrial respiratory chain complex I;^#^Coiled Coil (CC) or Toll-Interleukin Receptor (TIR)-Nucleotide Binding Site (NBS)-Leucine Rich Repeat (LRR);^+^nucleotide-binding (NB) motif-containing domain shared by human APAF-1, certain plant *R* gene products, and *Caenorhabditis elegans* CED-4 (ARC)

Among the genes predicted to be copy number reduced according to our array-CGH results, we observed an overrepresentation of biotic defence functions (see Fig. 6, Table 2C). This is an interesting observation with respect to the proposed ecological role of metal hyperaccumulation in plants (see Discussion). Almost all biotic stress-related genes predicted to be copy number reduced in *A. halleri* encode members of large protein families typically involved in plant innate immunity and designated as disease *Resistance (R)* genes, such as the predominating TIR-NBS-LRR receptor kinases (see Table 2C). Infecting pathogens generate characteristic molecular patterns that can be specifically recognized by cognate *R* gene products, which subsequently trigger a localized cell death response that is essential for plant disease resistance. The enrichment of *R* gene-related biotic stress functions among genes reduced in copy number in the hyperaccumulator *A. halleri* supports the elemental defence hypothesis as well as the trade-off hypothesis for the evolutionary role of elemental defence. Accordingly, elemental defences through metal hyperaccumulation allow for the loss of *R* genes, thus alleviating the fitness costs associated with *R* gene expression.

At lower levels of the ontological hierarchy, post-translational modification functions, and genes encoding glycine-rich and crinkly-like proteins were over-represented among the copy number expanded genes of *A. halleri*, whereas DC1 domain-containing, as well as protein degradation/E3-SCF-F-box and cysteine protease functions were significantly enriched among the genes predicted to be copy number reduced. Furthermore, genes predicted to be copy number reduced in both *A. halleri* and *A. lyrata* according to our array-CGH, showed a significant enrichment of the MapMan functional category or BIN “DNA.synthesis/chromatin structure.retrotransposon/transposase” (20.4% in *A. halleri*, 38.7% in *A. lyrata*; 1.5% of all genes on array; data not shown). Contrary to this observation, it is known that transposable element gene families have undergone an expansion in the *A. lyrata* genome relative to *A. thaliana* [35].

### Contributions of segmental duplications or deletions to copy number divergence

**Fig. 7:**
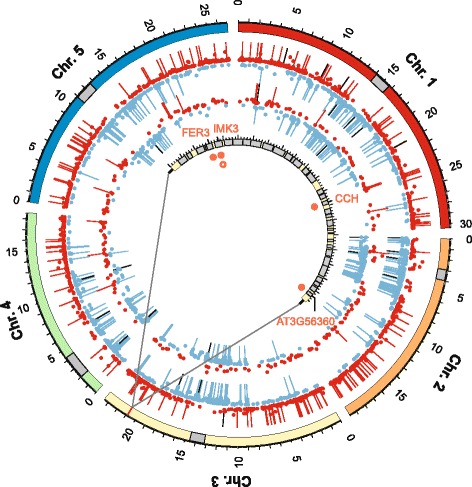
Genomic distribution of copy number alterations in *A. halleri* and *A. lyrata*, relative to the *A. thaliana* genome. The five chromosomes of *A. thaliana* are represented in different colours, with centromeric regions shaded in *grey* and radial axis labels indicating genomic position in Mbp. *Round* symbols mark the positions of genes found to be copy number expanded (*red*) and reduced (*blue*), respectively, in *A. halleri* (*outer concentric ring*) and *A. lyrata* (*inner concentric ring*), with radial positions scaled to the *L* *o* *g*_2_ signal ratio of the heterologous species vs. *A. thaliana*. Line symbols mark the positions of 20-kb genomic regions, within which a minimum of 5 kb region is found to be copy number expanded (*red*) or reduced (*blue*), respectively. *Black line* symbols mark positions of at least 3 consecutive concordant *red* or *blue lines*, corresponding to 40-kb genomic regions, in which a minimum of 10 kb is found to be copy number expanded or reduced. The centre of the radial plot shows a zoomed 0.1-Mbp region (chromosome 3, 20.8 to 20.9 Mbp), with red circles representing copy number expanded genes in *A. halleri* (*filled circle*) and *A. lyrata* (*open circle*), labelled by short gene names or AGI codes. Genes (*grey*) and intergenic regions (*pale yellow*) are marked; tickmarks are spaced by 1 kb

Next we tested whether large segmental duplications or deletions make major contributions to CND from *A. thaliana* in *A. halleri* or *A. lyrata*. We detected only few large segmental duplications (5 in *A. halleri* and 1 in *A. lyrata*) and deletions (12 in *A. halleri* and 7 in *A. lyrata*) comprising more than 10 kb (Fig. 7), by using a sliding window approach and the set parameters (see Methods). A total of six predicted segmental deletion regions, located in positions corresponding to *A. thaliana* chromosomes 1 to 4, were shared between *A. halleri* and *A. lyrata*. Most detected segmental deletions were located in or around regions corresponding to *A. thaliana* centromers. These segmental deletions were composed almost entirely of transposable element genes [56] and are thus likely to include regions of low sequence conservation rather than segmental deletions (see above). The sequences of centromeric regions are known to be highly divergent in both nucleotide composition and length even between closely related species [57]. None of the MapMan functional classes of top level ontological hierarchy that were significantly enriched among copy number divergent genes (see Fig. 6) was found similarly enriched among the genes contained in segmental duplications or deletions. Overall, CNDs, and especially CNEs, appeared to be dispersed evenly in the genomes of both *A. halleri* and *A. lyrata* (Fig. 7). A survey of highly conserved duplications in the human genome of 90 to 98% nucleotide sequence identity and > 1 kb in length revealed a similar pattern. Only one-third of duplicated genes were found in clusters or segments larger than 10 kb, whereas the remaining two-thirds were found dispersed in the euchromatic, i.e. gene-rich, regions all over the genome [58]. Our results suggest that copy number divergence between either *A. halleri* or *A. lyrata* and *A. thaliana* is the result of a large number of small-scale events and not of a small number of events affecting large genomic regions.

## Discussion

CNV detection pipelines employing arrays are still generally considered to be more accurate than sequencing-based algorithms [59–61], particularly for the detection of large duplicated segments or duplications of high sequence conservation [60, 61]. De novo assembly of non-model genomes often collapses paralogous gene copies into a single locus. While the traditional shotgun assembly resulted in a pronounced under-detection of highly identical gene CNV regions of sizes > 1 kb [58], de novo assembly of short reads missed 99% [60] of all known sequence duplications [62]. By contrast, the higher the similarity between paralogs, the higher is the probability of their detection by hybridization-based methods. This makes array-CGH methods particularly useful for detecting recent gene duplications or gene copy number divergence in emergent species. Recent and thus almost identical gene duplicates are expected to underlie the distinctive traits of species after short divergence times of 4 to 6 Mya, such as human and apes, or *A. halleri* and *A. thaliana*, as in our study [63]. Cross-species array-CGH methods thus fill a gap in the identification of gene CNVs and should be considered an important complementary approach to other CNV detection methods and for genome annotation.

Previously, array-CGH techniques were demonstrated to be quick, reliable and cost-effective in the detection of CNVs within the same species or lineage. Although whole genome tiling arrays are most widely used for this purpose, cross-species comparative genomic studies have also used gene expression arrays to compare the coding portions of genomes, for example the identification of genomic islands of speciation in three diverging populations of *Anopheles gambiae* [64] and the identification of human-specific gene duplications and contractions at a genome-wide level [65, 66]. The use of expression arrays for determining inter-species genic copy number divergence *via* array-CGH was generally demonstrated to give reliable and reproducible results [41, 45]. To date, Affymetrix ATH1 microarrays have been used in cross-species genomic DNA hybridizations for selecting a subset of probes for use in subsequent cross-species transcriptomics studies, but not with the aim to determine CNVs [67]. Conversely, combined with the global scaling approach developed here, array-CGH data could be used for adjusting hybridization signals in cross-species transcriptomics in order to improve accuracy without the loss of probe information. By comparison to previously published studies, besides being the only one in plants, our study decidedly benefits from the choice of species that are closely related to the reference species.

Our method yields erroneous results when the probes on ATH1 gene chip have been sourced from genomic regions prone to high sequence divergence such as transposable elements. We found an enrichment of retrotransposon/transposase functional category in the CNRs, contrary to the known expansion of TE families in *A. lyrata* relative to *A. thaliana*. We calculated an average of 39.1% sequence divergence between the ATH1 probe sequences corresponding to *A. thaliana* transposable element genes and the corresponding sequences of the *A. lyrata* reference genome, identified as the best blast hits. We thus propose that our result can be explained by the high degree of sequence divergence of transposable element gene families from *A. thaliana* in both heterologous species, as they diverged from *A. thaliana* at approximately the same time. Indeed, other sequence based orthology methods like Ensembl-Compara also report the TE genes as missing orthologs in *A. lyrata* [68]. Prior to the publication of the *A. lyrata* genome sequence, several population genomic studies predicted a reduction in TE copy number in comparison to *A. thaliana* [69, 70]. Similarly, a well-known CNE gene in *A. halleri* - *HMA4* was not detected by our methodology. On the ATH1 GeneChip, probe sequences for *HMA4* are located within the 3-´region of the *HMA4* coding sequence, which encodes the C-terminal cytoplasmic regulatory domain of the protein and is highly divergent in *A. halleri* (22% nucleotide sequence divergence compared to *A. thaliana*). This unusually high nucleotide sequence divergence explains our false negative result for *HMA4* copy number expansion. Additionally, a significant enrichment of genes “Not assigned” were found among copy number reduced genes of both *A. halleri* and *A. lyrata*. Among our predictions of CNRs common to both heterologous target species, we would generally expect false positives corresponding to genes that are highly divergent from their *A. thaliana* orthologues and that belong to fast-evolving groups of genes.

The sensitivity of our method for copy number reductions (CNRs) also appears to be lower than other methods but this is because employing the procedures of both previous studies resulted in the prediction of a substantial, more than 26-fold excess of gene copy number reductions or deletions (Additional file 6) when compared to the high-confidence set of predictions shared by both Ensembl Plants [68] and the *A. lyrata* reference genome project [35]. Thus, specifically accounting for inter-species sequence divergence in the analysis of cross-species array-CGH data enhances accuracy and prevents the under-reporting of true gene orthologs. We also note that our superior results in comparison to previous methods might reflect the fact that each method is most suited to the array platform and scientific goal for which it was developed. For example, single base substitutions are likely to be less deleterious in hybridizations to 500-bp probes on spotted cDNA arrays [43] than to the much shorter 25-nt probes of the ATH1 array. Different from the ATH1 array, tiling arrays do not require any summarization of signals from single probes within a probeset [42].

Extrapolating from our results that several genes known to be highly-expressed in *A. halleri* relative to *A. thaliana* are also copy number expanded, there might be a general association of gene copy number expansion with enhanced transcript levels. Indeed, one of the ways in which gene copy number divergence can confer an adaptive advantage is by effecting changes in gene product dosage, with a high probability of generating a physiological effect. In support of this, drastic phenotypes have been demonstrated to result from modification of transcript levels resulting from alterations in gene copy number [17, 71–73]. Regarding the enrichment of mitochondrial electron transport functions in CNE genes of *A. halleri*, a function of mitochondrial proteins in metal hypertolerance or hyperaccumulation has not been reported to date, but complex III of the mitochondrial electron transport chain was proposed to be a major molecular target for Cd toxicity in plants [74]. Alternatively, our observation may relate to metabolic alterations allowing enhanced dicarboxylate accumulation. Malate and citrate have been identified as quantitatively important metal chelators in hyperaccumulator plants [75, 76], thought to operate mostly inside vacuoles and in the apoplastic xylem. An important role of these anions at storage sites might be supplying a charge balance rather than chelation. Finally, these copy number expansions could reflect a need for enhanced mitochondrial respiration to energize processes involved in metal hyperaccumulation or hypertolerance. Future experiments will have to address these alternative hypotheses on the possible roles of copy number expanded genes associated with mitochondrial electron transport and ATP synthesis in metal hypertolerance or hyperaccumulation or other traits of *A. halleri*.

The biotic defense functions were found enriched in CNR genes of *A. halleri*. The elemental defence hypothesis postulates that the extraordinarily high concentrations of metals accumulated in leaves of metal hyperaccumulator plants act as a defence against herbivory or pathogen attack [36, 37]. Furthermore, the trade-off hypothesis postulates that metal hyperaccumulation-based defences allow plants to reduce the production and thus fitness costs of secondary metabolite or protein-based organic defences, incurred through their direct metabolic expense or indirect metabolic expense in scarce nutrients such as sulfur and nitrogen [36]. A quantitative comparison of metabolic expenses of organic and elemental defences, however, has not been possible to date. Indeed, secondary metabolism-related functions were not found to be enriched among copy number reduced genes of *A. halleri* in this study. Thus, our results do not provide support for the commonly proposed metabolic cost trade-off hypothesis regarding elemental defences in hyperaccumulators.

A fitness cost of up to 9% was reported for the expression of a single *R* gene, *RPM1*, in *A. thaliana* [77]. Recent work has identified plant *R* genes as an important group of genes underlying instances of hybrid necrosis [78, 79]. *A. halleri* is an obligate outcrosser, for which post-zygotic hybrid incompatibility is expected to have more severe consequences than for its largely selfing relative *A. thaliana*. In metal hyperaccumulator plants, fitness costs of *R* gene expression could additionally involve the recently proposed inadvertent activation of R protein-mediated defence signalling by internal heavy metals [36]. The loss of *R* genes in *A. halleri* can be interpreted to suggest that for a plant capable of metal hyperaccumulation, threats from biotic stress are reduced on soils permitting the hyperaccumulation of heavy metals [80–82]. Our results thus suggest that CNV resulting from structural mutations are important for plant evolution occurring upon interaction with both biotic stress and abiotic environmental factors. Genetic alterations similar to those reported here for *A. halleri* might thus underlie other reports of niche adaptation and consequent speciation driven by herbivore pressure alone, or in combination with soil edaphic factors [80, 81].

## Conclusions

In this work, we generated conservative estimates of genome-wide between-species gene copy number divergence in two close relatives of the model plant *A. thaliana*, based on cross-species microarray hybridization data. We devised a novel normalization strategy, which requires less existing sequence data, yields more comprehensive results and performs better than previously developed methods. Functions in transition metal homeostasis were found to be most highly enriched among copy number expanded genes in the metal hyperaccumulator *A. halleri*, but not in the non-accumulating sister-species *A. lyrata*, despite a shared overall genome structure and an equivalent evolutionary divergence from *A. thaliana*. In combination with the enrichment of biotic stress functions among copy number reduced genes of *A. halleri* also observed here, our finding lends support to the elemental defence hypothesis for the evolution of the metal hyperaccumulation trait [37]. Our results highlight the genome-wide importance of gene copy number alterations in adaptive evolution and suggest that genome scans for copy number divergence can identify functional networks that have been targets of natural selection. We propose that our findings are applicable in ecotoxicology for identifying the types and targets of environmental change-mediated stress in suitable indicator organisms. Finally, our study has identified novel candidate genes for the future improvement of the molecular physiological understanding of metal hyperaccumulation and associated hypertolerance in plants.

## Methods

### Plant material

Two samples of leaf material of *Arabidopsis halleri* ssp. *halleri* (accession Langelsheim) were obtained, respectively, from one cloned individual (W 504) [30], and from 10 pooled F1 progeny grown from seeds of a controlled, reciprocal crosses between two Langelsheim individuals (Lan3.1 and Lan5) [31]. *Arabidopsis lyrata* ssp. *petrea* (accession Kubova Hut, kindly provided by Marc Macnair, University of Exeter) and *Arabidopsis thaliana* (accession Col-0) were grown from seed, and leaf material was pooled from 10 individuals, respectively. *A. thaliana* was cultivated on standard soil, and the other two species were cultivated hydroponically [31].

### Genomic DNA isolation

Genomic DNA was isolated, fragmented and end-labelled with Bio-N^6^-ddATP according to Borevitz et al. [83], with some modifications of the procedure. Total genomic DNA was isolated from 4 to 6 g fresh biomass of plant leaf tissue with cetyl trimethylammonium bromide (CTAB) buffer (0.8% (w/v) CTAB, 800 mM NaCl, 1% (w/v) N-laurylsarcosine, 140 mM sorbitol, 22 mM EDTA, 220 mM Tris pH 8). Frozen plant tissue was ground to a fine powder in liquid N_2_, transferred to a 50 ml tube containing 30 ml CTAB buffer and incubated at 65 °C with occasional vigorous shaking for 20 min. After addition of 12 ml chloroform/isoamylalcohol (24:1) and vigorous mixing, tubes were placed at room temperature (RT) on an inverter for 20 min. After centrifugation in a table-top centrifuge at 3,700 g for 5 min, the aqueous phase was transferred to a fresh tube, 1 vol. isopropanol was added and nucleic acids were precipitated on ice for 30 min. After centrifugation at 9,500 rpm (rotor: JA25-50), 4 °C for 8 min, the supernatant was drained and the pellets were resuspended in 6 ml ultrapure H_2_O. 1 vol. 4 M LiAc was added and the samples were incubated on ice for 20 min to precipitate RNA. After centrifugation at 9,500 rpm (rotor: JA25-50), 4 °C for 11 min, the supernatant was transferred to a fresh tube, 2 vol. ethanol were added and the samples were placed at RT for a few seconds. To collect the precipitate, samples were centrifuged at 12,000 rpm (rotor: JA25-50), 4 °C for 20 min. Pellets were resuspended in 1.35 ml ultrapure H_2_O, followed by the addition of 150 μl 3 M sodium acetate. Each sample was then split between two 2-ml eppendorf vials. One vol. of phenol/chloroform/isoamylalcohol (25:24:1) was added, vial contents were mixed by shaking, and subsequently centrifuged in a tabletop microcentrifuge at 14,000 rpm (> 16,800 g) for 5 min to resolve phases. The aqueous phase was collected, 2 vol. ethanol were added and vials were placed on ice for 5 min. The precipitate was collected by centrifugation in a tabletop microcentrfuge at 14,000 rpm (> 16,800 g) for 5 min. Each pellet was washed with 80% (v/v) ethanol and dried at 37 °C for 10 min. Pellets were resuspended by gentle pippetting and combined for each sample in 300 μl ultrapure H_2_O. Nucleic acid concentration and purity was determined spectrophotometrically by measuring absorption at 260 and 280 nm and by subjecting 4 *μ*g DNA of each preparation to agarose gel electrophoresis.

### Genomic DNA fragmentation, 3’-end labelling with biotin and microarray hybridization

Nucleic acid (600 μg) were digested with 10 U RNase (RNase ONE Ribonuclease, Promega) in 500 μl total volume at 37 °C for 15 min. After RNase digest, 50 μl 3 M sodium acetate were added to the samples, followed by extraction with 1 vol. phenol/chloroform/isoamylalcohol. The aqueous phase was collected, 2 vol. ethanol were added and samples were placed on ice for 5 min. The precipitate was collected by centrifugation in a tabletop microcentrifuge at 20,800 g, 4 °C for 15 min. The pellet was washed with 80% (v/v) ethanol and dried. Pellets were resuspended in 200 μl ultrapure H_2_O by gentle pipetting. Genomic DNA concentration and purity were determined spectrophotometrically by measuring absorption at 260 and 280 nm and by subjecting 1 μg of each preparation to agarose gel electrophoresis. 20 μg of genomic DNA were fragmented with 0.33 U DNase (RQ1 RNase-free DNase, Promega) for 4 min at 37 °C, in a reaction containing 1x One-Phor-All Buffer Plus (Amersham Biosciences) and 1.5 mM CoCl_2_ in a total volume of 35 μl. To ensure an equal start and length of all reactions, the DNase was placed in a small drop at the inner wall of each reaction tube and spun down into the reaction mix. All reactions were briefly vortexed and spun down before they were incubated at 37 °C in a water bath. Immediately after the digest, the DNase was heat-inactivated at ≥ 95 °C for 15 min. After heat-inactivation, the reaction tubes were placed on ice. DNA digestion was confirmed by subjecting 3 μl of a reaction to DNA-gel electrophoresis on a 2% (w/v) agarose gel. Oligonucleotides of 22 and 50 bp length were used as size markers. A broad band indicated good digestion with fragments between 20 and 50 bp. The genomic DNA fragments were 3^′^-end labelled with biotin by adding 40 U terminal deoxynucleotidyl transferase (Promega) and 2 μl of 1 mM Bio-N^6^-ddATP (Enzo Life Sciences, USA) to the remaining 32 μl of the fragmentation reaction and incubating the samples at 37 °C in the dark for 1 h. Hybridization was conducted according to the standard protocol for hybridizing fragmented cRNA to the Affymetrix ATH1 GeneChip [31], using 20 μl of the fragmented and labelled genomic DNA instead of fragmented cRNA. Hybridized arrays were washed and stained in an Affymetrix Fluidics Station FS450, and the fluorescent signals were measured with an Affymetrix GeneChip Scanner 3000 using standard protocols provided by the manufacturer.

### Reference datasets for *A. halleri* and *A. lyrata*

ATH1 GeneChip^®;^ microarray contains between 11 and 20 probe pairs per gene in a probeset, each probe comprising 25 nucleotides, for each of 23,725 *A. thaliana* genes. Because of the design of the ATH1 microarray for genome-wide quantification of transcript levels in *A. thaliana*, all probes correspond to transcribed regions of the *A. thaliana* genome. The available sequences of 39 nuclear-encoded cDNAs of *A. halleri* ssp. *halleri*, accession Langelsheim, were used to assess the effect of nucleotide sequence divergence of heterologous species on signal intensities when hybridizing to probes designed for *A. thaliana*. After removing known copy number expanded genes of *A. halleri* (Additional file 5), 33 genes/probesets with a total of 273 probe sequences on the ATH1 GeneChip served as a representative *A. halleri* reference dataset (Additional file 1). We generated a corresponding reference dataset for *A. lyrata*, exploiting the sequence information from the *A. lyrata* reference genome [35]. We generated a custom BLAST database (ALyDB) from the fasta sequences of the FilteredModels6 gene models track from the *A. lyrata* genome assembly version1. Individual probe sequences from the ATH1 GeneChip were blasted (nucleotide-short) against the ALyDB database with default parameters to report a maximum of 3 best alignments. Out of a total of 22,810 probe sets on the ATH1 GeneChip, we retained 16,626 probe sets that reported the top hit in the same *A. lyrata* gene for at least 8 of the probe sequences of a given probe set. We then removed probesets mapping to non-nuclear genomic sequences, and we randomly selected 44 probesets corresponding to single-copy genes based on the available information [68], to serve as representative *A. lyrata* reference dataset (Additional file 1). The numbers and positions of mismatches between probe sequences and the corresponding sequences of the heterologous target species, *A. halleri* and *A. lyrata*, were recorded for both reference datasets (Additional file 2, Additional file 1) in order to determine their effects on hybridization signal intensity in array-CGH.

### Assessing and adjusting for sequence divergence in probe sequence hybridization

Upon cross-species hybridization, there can only be one of two possible outcomes for each nucleotide position of the probe: match (hybridization) or mismatch (no hybridization). Given that each probe is 25 nucleotides long, cross-species hybridization can be considered as a series of Bernoulli trials, and thus the distribution of mismatches in a 25 nucleotide long probe can be captured by a Binomial distribution. Published data suggested that the coding sequences of *A. halleri* and *A. thaliana* exhibit on average 94% identical nucleotides [30, 31] (Additional file 1), and thus, the probability of occurrence of a mismatch between the heterologous target ssDNA and the corresponding probe sequence on the microarray is 0.06 [30]. Thus, the expected number of mismatches in a single probe sequence is the binomial probability *P* of *k* number of mismatches occurring in a probe of length *n*. This can be calculated by the following formula where, *p*= probability of mismatch, *k* = number of positions with mismatch (between 0 and 25), *n*= length of probe (25 for probe sequences on ATH1 Gene Chip). We computed signal correction factor *S*_*k*_ as the arithmetic mean of the inverse ratios of hybridization signal intensity *I*_*ik*_ from probe sequence *i* containing *k* mismatches in heterologous species *A. lyrata /A. halleri* to the corresponding hybridization signal intensity *H*_*ik*_ for the homologous target species *A. thaliana*:

1 $$ S_{k} = \frac{1}{N_{k}} \sum\limits_{i=1}^{N_{k}} \frac{H_{ik}} {I_{ik}}  $$

where *N*_*k*_ is the number of probes containing *k* mismatches. To calculate the global scaling factor *S*, took the mean of signal correction factors *S*_*k*_ weighted by their expected frequency *P*_*n*_(*k*), for target probes containing *k*= 0 to 4 mismatches.

2 $$  S = \sum\limits_{k=0}^{4}S_{k}.P_{n}(k)  $$

### Evaluation of normalization methods

To choose the most appropriate normalization method, we used a dataset of 14 *A. halleri* genes for which the gene copy number is reliably known (Additional file 5) through genomic DNA blots (11 genes, [31]) or taken from literature (2 genes, [18, 38]). The fact that this set of genes is composed primarily of metal homeostasis genes does not bias the validation, because both single-copy genes and copy number expanded genes are represented equally. Out of these 14 genes, 6 are copy number expanded and 8 are present as single copies. We evaluated normalization procedures involving combinations of Quantile [48] and VSN [84] normalization algorithms, MAS5 [85] and GCRMA [86] background corrections and two significance tests — ANOVA (Limma package in R [87]) and Wilcoxon rank sum test, for each gene. The global scaling factor described above was calculated after each normalization procedure and applied to the hybridization data of *A. halleri*. Subsequently, we compared the sensitivity (percentage of true positives correctly identified), specificity (percentage of true negatives correctly identified) and precision (percentage of true positives out of all predicted positives) of each procedure, based on the number of genes in Additional file 5 for which copy number state was correctly estimated. We found the combination of MAS5 background correction and VSN normalization, followed by ANOVA significance test at the probe level to perform the best (66.7% sensitivity, 87.5% specificity). It was twice as sensitive as the second best normalization method GCRMA (33.3% sensitivity) while maintaining the same specificity.

### Normalization, scaling and estimating genic CNVs

The raw intensity (.CEL format) files from affymetrix GeneChip^Ⓡ^ ATH1 genome array were loaded into R (version 2.14.1). One of the *A. halleri* gDNA hybridizations (Mw_101105_03X.CEL) was detected to have some compact defects affecting nearly 2% of the total probes. This defect was corrected using Harshlight package in R [88], wherein the corresponding probe signal of the replicate GeneChip replaced the values of erroneous probes. We used the affy [89] and VSN2 [84] package to apply MAS5.0 [85] background correction followed by masking of probes from mitochondrial, chloroplast, control and known multi-copy genes in *A. halleri*. VSN normalization was applied to the replicate array hybridizations of each species separately. We scaled the signals of the entire *A. halleri* and *A. lyrata* gDNA hybridized GeneChips by their respective global scaling factor *S*, as calculated above, to make the hybridizations comparable. Normalized and scaled data were then subjected to an ANOVA test at individual probe level (lmFit and eBayes functions of Limma package [87]) to reliably identify genes with differential hybridization signal between species. Genes with *L* *o* *g*_2_ ratio ≥ 1 of scaled signal intensities in *A. halleri* or *A. lyrata*, respectively, *vs. A. thaliana* and corrected *P*-value ≤ 0.1 (Benjamini-Hochberg multiple testing correction [90]) were considered candidates for copy number expansion in the heterologous species. Similarly, genes with *L* *o* *g*_2_ ratio ≤ -1 and corrected *P*-value ≤ 0.1 (Benjamini-Hochberg multiple testing correction) were considered candidate genes exhibiting copy number reduction in the heterologous species (complete dataset provided in Additional file 9). The log-ratio thresholding employed here is a commonly used robust method of determining CNVs from array-CGH data [45] to which we have added the power of statistical analysis.

### Construction of *A. lyrata* reference datasets

To construct an *A. lyrata* reference dataset for comparison with the CNDs predicted by our array-CGH approach, we first retrieved copy number information for each *A. lyrata* gene ortholog relative to the corresponding *A. thaliana* gene from two sources. The first source were Ensembl Plants orthology predictions (http://plants.ensembl.org; ref. [68]), and the second source was the *A. lyrata* genome sequencing project [35]. In the *A. lyrata* genome sequencing project, orthology classifications between *A. thaliana* and *A. lyrata* genes were established through reciprocally best BLAST hits (personal communication, Dr. Tina T. Hu, Lewis-Sigler Institute for Integrative Genomics, Princeton University USA), predicting 1,522 copy number expanded genes and 826 copy number reduced genes (Additional file 6). Only about one third of the predicted CNEs (384 genes) were common to both reference-genome based predictions (Additional file 6). We chose the Ensembl Plants predictions as our primary basis for validation of our array-CGH results because they were based on a benchmarked and robust orthology prediction pipeline [68].

### Reproduction of two alternative array-CGH methods for comparison

Two previously published methods for cross-species array-CGH based prediction of gene copy number divergence were applied to our gDNA hybridization data. Machado and Renn [43] used spotted cDNA arrays with 500 bp long probes. In contrast, we employed the Affymetrix ATH1 GeneChip with ∼22,500 features, each comprising a set of usually 11 probes (25 bp long). This necessitated some minor modifications to the original workflow, as outlined below. Starting with raw hybridization signals from *A. lyrata* and *A. thaliana*, we performed background correction by MAS instead of the *“minimum”* algorithm, because *minimum* is specific for use with two-colour hybridization arrays and MAS background correction appears most similar in approach to *minimum*. Thereafter, within-array normalizations were carried out using a subset of 1,000 conserved genes. For this, we generated a list of 8,268 *A. lyrata* single-copy orthologs that show ≥ 95% sequence identity with *A. thaliana*. Out of the probesets representing these genes, we then randomly picked a subset of 1,000 probesets. We applied loess normalization [91] based on perfect match (PM) probe hybridization signal intensities of these 1,000 conserved genes. Summarization of probeset intensities to a single value was done using the *Avdiff* algorithm [92]. A linear model was fitted to the data using lmFit and eBayes functions in the Limma package from R [87]. *P*-values were calculated (Multiple testing correction by FDR). Genes represented by probesets displaying *L* *o* *g*_2_ ratio > 0 of normalized signal intensities for *A. lyrata vs. A. thaliana* and *P*-value ≤ 0.1 were classified as duplicated or copy number expanded. Similarly, genes represented by probesets displaying a *L* *o* *g*_2_ ratio < 0 of normalized signal intensities for *A. lyrata vs. A. thaliana* and *P*-value ≤ 0.1 were classified as deleted or copy number reduced.

We also implemented an approach analogous to Darby et al. [42] used Affymetrix *Caenorhabditis elegans* tiling arrays with probes of 25 nucleotides in length. In brief, this involves normalization at the probe level by adjusting for thermodynamic binding affinity, and then a species-specific scaling based on the array control probes that are expected to be equally dissimilar in all species. Thus, we first calculated the thermodynamic binding affinity — *Δ* *G*37 value (Gibb’s free energy estimate by Santa-Lucia model [93]) for each probe on the ATH1 GeneChip using the oligoprop package in Matlab. We plotted the background corrected (MAS) signal intensities of the custom control probes against their thermodynamic binding affinities to obtain the model parameters (intercept and error terms) that are required to normalize probe signal intensities for all the probes on the chip in a species-specific manner. Subsequently, the median signal intensity of the control probes of the hybridized target species was used to scale the hybridization signal intensities of all probes on the ATH1 GeneChip. Darby et al. performed a visual confirmation of 131 known genomic deletions for their data. This was not possible for us because we expected thousands of gene duplications/deletions in *A. lyrata* (Additional file 6). Consequently, we summarized the probe *L* *o* *g*_2_ ratios of signal intensities for each *A. lyrata* gene relative to *A. thaliana* by computing probeset means from the constituent probes. Then, in a conservative implementation of the procedure of Darby et al., a gene with *L* *o* *g*_2_ ratio of ≥ 0.25 was classified as a copy number expanded gene and a gene with *L* *o* *g*_2_ ratio of ≤−0.25 was classified as a gene copy number reduction in *A. lyrata* relative to *A. thaliana*.

### MapMan functional enrichment analysis

We carried out an over-representation analysis (ORA) based on the MapMan functional ontology [51] to detect functional enrichment in the genes with CNVs by comparison to all nuclear genes represented on the array. We chose this ontology because it is plant-specific, has been curated by experts in the constituent subject areas and uniquely includes functional categories of specific relevance for plant biology. The MapMan ontology is hierarchical, with 36 major functional categories called BINs at the top level; we used only these top BINs for ORA. The top level of MapMan bears similarity to the generic Gene Ontology (GO) slim [94], widely used for summarizing GO annotations in microarrays. The Affymetrix ATH1 probeset identifiers were used as primary gene identifiers to avoid redundancy in assigning genes to their functional categories (BIN). Given a list of copy number divergent genes, we calculated the percentage of genes in the list assigned to a particular function (BIN) and compared it to the overall percentage of genes with the same function present on the ATH1 GeneChip, applying Fisher’s exact test [95] to test for statistically significant differences (Benjamini-Hochberg corrected *P*-value ≤ 0.05). The annotation of genes known or purported to be involved in transition metal homeostasis functions was sourced from an expert-curated list. This list represents an updated version of the MapMan functional category “metal handling”, which had originally been generated by Ute Krämer, but was found to exclude some genes that were functionally characterized only recently and to contain several genes based on previous misannotations. The updated list is provided in Additional file 8 and will be submitted to the curators of MapMan for updating their current metal homeostasis gene annotations.

### Regions enriched in genic copy number divergence

To assess the contributions of large-scale structural rearrangements to copy number divergence, we implemented a sliding window approach, in which a 20 kb long window was moved along each chromosome of *A. thaliana* with 10 kb step sizes and scored positive upon the presence of a CND region covering at least 5 kb of contiguous genes with concordant CND or CNR calls, respectively, in *A. halleri* or *A. lyrata*. A large CND region was defined as three consecutive windows with positive and concordant calls for either CNE or CNR, which would ensure a minimum of 10 kb to be copy number expanded or reduced in a stretch of 40 kb.

## Additional files

Additional file 1 Representative reference datasets. Summary of number and position of sequence-probe hybridization mismatches relative to *A. thaliana* gene sequence. (XLSX 73 kb)

Additional file 2 Mismatches with respect to ATH1 GeneChip probe sequences in representative reference datasets of *A. halleri* and *A. lyrata*. (XLSX 47 kb)

Additional file 3 List of copy number expanded and copy number reduced genes in *A. halleri* and *A. lyrata*, by comparison to *A. thaliana*. (XLSX 1331 kb)

Additional file 4 Gene copy number expansions (CNEs) and reductions (CNRs) in *A. halleri* relative to *A. thaliana* generated for two datasets, each comprising one of the two *A. halleri* hybridizations and its in-silico replicate displaying the same amount of within-sample variation as *A. lyrata* hybridizations. (PDF 23 kb)

Additional file 5 Gold standard dataset of genes of known copy number in *A. halleri*. (XLSX 40 kb)

Additional file 6 Numbers of genes predicted to exhibit copy number divergence in *A. lyrata* by comparison to *A. thaliana*. (XLSX 46 kb)

Additional file 7 Validation of array-CGH results against all genes predicted to be **(A)** copy number expanded (CNEs) and **(B)** copy number reduced (CNRs) in *A. lyrata* (see Table 1). (XLSX 43 kb)

Additional file 8 Manually curated list of MapMan transition metal homeostasis genes of *A. thaliana*. (XLSX 44 kb)

Additional file 9 Complete dataset expanded from Additional file 3. (XLS 16282 kb)

## References

[1] Clark RM, Schweikert G, Toomajian C, Ossowski S, Zeller G, Shinn P, Warthmann N, Hu TT, Fu G, Hinds DA, Chen H, Frazer KA, Huson DH, Schölkopf B, Nordborg M, Rätsch G, Ecker JR, Weigel D (2007). Common sequence polymorphisms shaping genetic diversity in Arabidopsis thaliana. Science (New York).

[2] Lister R, O’Malley RC, Tonti-Filippini J, Gregory BD, Berry CC, Millar AH, Ecker JR (2008). Highly integrated single-base resolution maps of the epigenome in Arabidopsis. Cell.

[3] Cokus SJ, Feng S, Zhang X, Chen Z, Merriman B, Haudenschild CD, Pradhan S, Nelson SF, Pellegrini M, Jacobsen SE (2008). Shotgun bisulphite sequencing of the Arabidopsis genome reveals DNA methylation patterning. Nature.

[4] Becker C, Hagmann J, Müller J, Koenig D, Stegle O, Borgwardt K, Weigel D (2011). Spontaneous epigenetic variation in the *Arabidopsis thaliana* methylome. Nature.

[5] Schmitz RJ, Schultz MD, Lewsey MG, O’Malley RC, Urich MA, Libiger O, Schork NJ, Ecker JR (2011). Transgenerational Epigenetic Instability Is a Source of Novel Methylation Variants. Science.

[6] Greaves IK, Groszmann M, Ying H, Taylor JM, Peacock WJ, Dennis ES (2012). Trans chromosomal methylation in Arabidopsis hybrids. Proc Natl Acad Sci U S A.

[7] Hancock AM, Brachi B, Faure N, Horton MW, Jarymowycz LB, Sperone FG, Toomajian C, Roux F, Bergelson J (2011). Adaptation to climate across the *Arabidopsis thaliana* genome. Science (New York).

[8] Fournier-Level A, Korte A, Cooper MD, Nordborg M, Schmitt J, Wilczek AM (2011). A map of local adaptation in *Arabidopsis thaliana*. Science (New York).

[9] Innan H, Kondrashov F (2010). The evolution of gene duplications: classifying and distinguishing between models. Nat Rev Genet.

[10] Muñoz-Amatriaín M, Eichten SR, Wicker T, Richmond TA, Mascher M, Steuernagel B, Scholz U, Ariyadasa R, Spannagl M, Nussbaumer T, Mayer KF, Taudien S, Platzer M, Jeddeloh JA, Springer NM, Muehlbauer GJ, Stein N (2013). Distribution, functional impact, and origin mechanisms of copy number variation in the barley genome. Genome Biol.

[11] Swanson-Wagner RA, Eichten SR, Kumari S, Tiffin P, Stein JC, Ware D, Springer NM (2010). Pervasive gene content variation and copy number variation in maize and its undomesticated progenitor. Genome Res.

[12] Henrichsen CN, Chaignat E, Reymond A (2009). Copy number variants, diseases and gene expression. Hum Mol Genet.

[13] Perry GH, Dominy NJ, Claw KG, Lee AS, Fiegler H, Redon R, Werner J, Villanea FA, Mountain JL, Misra R, Carter NP, Lee C, Stone AC (2007). Diet and the evolution of human amylase gene copy number variation. Nat Genet.

[14] Van de Peer Y, Maere S, Meyer A (2009). The evolutionary significance of ancient genome duplications. Nat Rev Genet.

[15] Sutton T, Baumann U, Hayes J, Collins NC, Shi BJ, Schnurbusch T, Hay A, Mayo G, Pallotta M, Tester M, Langridge P (2007). Boron-toxicity tolerance in barley arising from efflux transporter amplification. Science (New York).

[16] Maron LG, Guimarães CT, Kirst M, Albert PS, Birchler JA, Bradbury PJ, Buckler ES, Coluccio AE, Danilova TV, Kudrna D, Magalhaes JV, Piñeros MA, Schatz MC, Wing RA, Kochian LV (2013). Aluminum tolerance in maize is associated with higher *MATE1* gene copy number. Proc Natl Acad Sci U S A.

[17] Hanikenne M, Kroymann J, Trampczynska A, Bernal M, Motte P, Clemens S, Krämer U (2013). Hard selective sweep and ectopic gene conversion in a gene cluster affording environmental adaptation. PLoS Genet.

[18] Hanikenne M, Talke IN, Haydon MJ, Lanz C, Nolte A, Motte P, Kroymann J, Weigel D, Krämer U (2008). Evolution of metal hyperaccumulation required *cis*-regulatory changes and triplication of *HMA4*. Nature.

[19] Santuari L, Pradervand S, Amiguet-Vercher AM, Thomas J, Dorcey E, Harshman K, Xenarios I, Juenger TE, Hardtke CS (2010). Substantial deletion overlap among divergent Arabidopsis genomes revealed by intersection of short reads and tiling arrays. Genome Biol.

[20] DeBolt S (2010). Copy number variation shapes genome diversity in Arabidopsis over immediate family generational scales. Genome Biol Evol.

[21] Hanada K, Zou C, Lehti-Shiu MD, Shinozaki K, Shiu SH (2008). Importance of lineage-specific expansion of plant tandem duplicates in the adaptive response to environmental stimuli. Plant Physiol.

[22] Dassanayake M, Oh DH, Haas JS, Hernandez A, Hong H, Ali S, Yun DJ, Bressan RA, Zhu JK, Bohnert HJ, Cheeseman JM (2011). The genome of the extremophile crucifer *Thellungiella parvula*. Nat Genet.

[23] Flagel LE, Wendel JF (2009). Gene duplication and evolutionary novelty in plants. New Phytol.

[24] Cannon SB, Mitra A, Baumgarten A, Young ND, May G (2004). The roles of segmental and tandem gene duplication in the evolution of large gene families in *Arabidopsis thaliana*. BMC Plant Biol.

[25] Hunter B, Bomblies K (2010). Progress and Promise in using Arabidopsis to study adaptation, divergence, and speciation. Arabidopsis Book.

[26] Bomblies K, Weigel D (2007). Arabidopsis: a model genus for speciation. Curr Opin Genet Dev.

[27] Krämer U (2010). Metal hyperaccumulation in plants. Annu Rev Plant Biol.

[28] Koch M, Haubold B, Mitchell-Olds T (2001). Molecular systematics of the Brassicaceae: evidence from coding plastidic *matK* and nuclear *Chs* sequences. Am J Bot.

[29] Beilstein MA, Nagalingum NS, Clements MD, Manchester SR, Mathews S (2010). Dated molecular phylogenies indicate a Miocene origin for *Arabidopsis thaliana*. Proc Natl Acad Sci U S A.

[30] Weber M, Harada E, Vess C, Roepenack-Lahaye EV, Clemens S, v Roepenack-Lahaye E (2004). Comparative microarray analysis of *Arabidopsis thaliana* and *Arabidopsis halleri* roots identifies nicotianamine synthase, a ZIP transporter and other genes as potential metal hyperaccumulation factors. Plant J.

[31] Talke IN, Hanikenne M, Krämer U (2006). Zinc-dependent global transcriptional control, transcriptional deregulation, and higher gene copy number for genes in metal homeostasis of the hyperaccumulator *Arabidopsis halleri*. Plant Physiol.

[32] Becher M, Talke IN, Krall L, Krämer U (2004). Cross-species microarray transcript profiling reveals high constitutive expression of metal homeostasis genes in shoots of the zinc hyperaccumulator *Arabidopsis halleri*. Plant J.

[33] Deinlein U, Weber M, Schmidt H, Rensch S, Trampczynska A, Hansen TH, Husted SR, Schjoerring JK, Talke IN, Krämer U, Clemens S (2012). Elevated nicotianamine levels in *Arabidopsis halleri* roots play a key role in zinc hyperaccumulation. Plant Cell.

[34] Dräger DB, Desbrosses-Fonrouge AG, Krach C, Chardonnens AN, Meyer RC, Saumitou-Laprade P, Krämer U (2004). Two genes encoding *Arabidopsis halleri* MTP1 metal transport proteins co-segregate with zinc tolerance and account for high *MTP1* transcript levels. Plant J Cell Mol Biol.

[35] Hu TT, Pattyn P, Bakker EG, Cao J, Cheng JF, Clark RM, Fahlgren N, Fawcett JA, Grimwood J, Gundlach H, Haberer G, Hollister JD, Ossowski S, Ottilar RP, Salamov AA, Schneeberger K, Spannagl M, Wang X, Yang L, Nasrallah ME, Bergelson J, Carrington JC, Gaut BS, Schmutz J, Mayer KFX, Van de Peer Y, Grigoriev IV, Nordborg M, Weigel D, Guo YL (2011). The *Arabidopsis lyrata* genome sequence and the basis of rapid genome size change. Nat Genet.

[36] Poschenrieder C, Tolrà R, Barceló J (2006). Can metals defend plants against biotic stress?. Trends Plant Sci.

[37] Boyd RS (2007). The defense hypothesis of elemental hyperaccumulation: status, challenges and new directions. Plant Soil.

[38] Shahzad Z, Gosti F, Frérot H, Lacombe E, Roosens N, Saumitou-Laprade P, Berthomieu P (2010). The five AhMTP1 zinc transporters undergo different evolutionary fates towards adaptive evolution to zinc tolerance in *Arabidopsis halleri*. PLoS Genet.

[39] Redman JC, Haas BJ, Tanimoto G, Town CD (2004). Development and evaluation of an Arabidopsis whole genome Affymetrix probe array. Plant J Cell Mol Biol.

[40] Bar-Or C, Bar-Eyal M, Gal TZ, Kapulnik Y, Czosnek H, Koltai H (2006). Derivation of species-specific hybridization-like knowledge out of cross-species hybridization results. BMC Genomics.

[41] Bar-Or C, Czosnek H, Koltai H (2007). Cross-species microarray hybridizations: a developing tool for studying species diversity. Trends Genet.

[42] Darby BJ, Jones KL, Wheeler D, Herman MA (2011). Normalization and centering of array-based heterologous genome hybridization based on divergent control probes. BMC Bioinforma.

[43] Machado HE, Renn SCP (2010). A critical assessment of cross-species detection of gene duplicates using comparative genomic hybridization. BMC Genomics.

[44] Gilbert LB, Chae L, Kasuga T, Taylor JW (2011). Array Comparative Genomic Hybridizations: assessing the ability to recapture evolutionary relationships using an in silico approach. BMC Genomics.

[45] Renn SCP, Machado HE, Jones A, Soneji K, Kulathinal RJ, Hofmann Ha (2010). Using comparative genomic hybridization to survey genomic sequence divergence across species: a proof-of-concept from Drosophila. BMC Genomics.

[46] Gilad Y, Rifkin SA, Bertone P, Gerstein M, White KP (2005). Multi-species microarrays reveal the effect of sequence divergence on gene expression profiles. Genome research.

[47] Wright SI, Lauga B, Charlesworth D (2003). Subdivision and haplotype structure in natural populations of *Arabidopsis lyrata*. Mol Ecol.

[48] Bolstad BM, Irizarry RA, Astrand M, Speed TP (2003). A comparison of normalization methods for high density oligonucleotide array data based on variance and bias. Bioinformatics (Oxford).

[49] Irizarry RA, Hobbs B, Collin F, Beazer-Barclay YD, Antonellis KJ, Scherf U, Speed TP (2003). Exploration, normalization, and summaries of high density oligonucleotide array probe level data. Biostatistics (Oxford).

[50] Lynch M (2010). Evolution of the mutation rate. Trends Genet.

[51] Thimm O, Bläsing O, Gibon Y, Nagel A, Meyer S, Krüger P, Selbig J, Müller LA, Rhee SY, Stitt M (2004). MAPMAN: a user-driven tool to display genomics data sets onto diagrams of metabolic pathways and other biological processes. Plant J.

[52] Ó Lochlainn S, Bowen HC, Fray RG, Hammond JP, King GJ, White PJ, Graham NS, Broadley MR (2011). Tandem quadruplication of *HMA4* in the zinc (Zn) and cadmium (Cd) hyperaccumulator *Noccaea caerulescens*. PLoS ONE.

[53] Willems G, Dräger DB, Courbot M, Godé C, Verbruggen N, Saumitou-Laprade P (2007). The genetic basis of zinc tolerance in the metallophyte *Arabidopsis halleri* ssp. *halleri* (Brassicaceae): an analysis of quantitative trait loci. Genetics.

[54] Song WY, Choi KS, Alexis DA, Martinoia E, Lee Y (2011). *Brassica juncea* Plant Cadmium Resistance 1 protein (BjPCR1) facilitates the radial transport of calcium in the root. Proc Natl Acad Sci U S A.

[55] Song WY, Choi KS, Kim DY, Geisler M, Park J, Vincenzetti V, Schellenberg M, Kim SH, Lim YP, Noh EW, Lee Y, Martinoia E (2010). Arabidopsis PCR2 is a zinc exporter involved in both zinc extrusion and long-distance zinc transport. Plant Cell.

[56] Wong LH, Choo KHA (2004). Evolutionary dynamics of transposable elements at the centromere. Trends Genet.

[57] Henikoff S, Ahmad K, Malik HS (2001). The centromere paradox: stable inheritance with rapidly evolving DNA. Science (New York).

[58] Eichler EE (2001). Segmental duplications: what’s missing, misassigned, and misassembled–and should we care?. Genome Res.

[59] Alkan C, Coe BP, Eichler EE (2011). Genome structural variation discovery and genotyping. Nat Rev Genet.

[60] Alkan C, Sajjadian S, Eichler EE (2011). Limitations of next-generation genome sequence assembly. Nat Methods.

[61] Teo SM, Pawitan Y, Ku CS, Chia KS, Salim A (2012). Statistical challenges associated with detecting copy number variations with next-generation sequencing. Bioinformatics (Oxford).

[62] Mills RE, Walter K, Stewart C (2011). Mapping copy number variation by population-scale genome sequencing. Nature.

[63] Dennis MY, Nuttle X, Sudmant PH, Antonacci F, Graves TA, Nefedov M, Rosenfeld JA, Sajjadian S, Malig M, Kotkiewicz H, Curry CJ, Shafer S, Shaffer LG, de Jong PJ, Wilson RK, Eichler EE (2012). Evolution of human-specific neural *SRGAP2* genes by incomplete segmental duplication. Cell.

[64] Turner TL, Hahn MW, Nuzhdin SV (2005). Genomic islands of speciation in *Anopheles gambiae*. PLoS Biol.

[65] Fortna A, Kim Y, MacLaren E, Marshall K, Hahn G, Meltesen L, Brenton M, Hink R, Burgers S, Hernandez-Boussard T, Karimpour-Fard A, Glueck D, McGavran L, Berry R, Pollack J, Sikela JM (2004). Lineage-specific gene duplication and loss in human and great ape evolution. PLoS Biol.

[66] Dumas L, Kim YH, Karimpour-Fard A, Cox M, Hopkins J, Pollack JR, Sikela JM (2007). Gene copy number variation spanning 60 million years of human and primate evolution. Genome Res.

[67] Hammond JP, Bowen HC, White PJ, Mills V, Pyke KA, Baker AJM, Whiting SN, May ST, Broadley MR (2006). A comparison of the *Thlaspi caerulescens* and *Thlaspi arvense* shoot transcriptomes. New Phytologist.

[68] Vilella AJ, Severin J, Ureta-Vidal A, Heng L, Durbin R, Birney E (2009). EnsemblCompara GeneTrees: Complete, duplication-aware phylogenetic trees in vertebrates. Genome Res.

[69] Wright SI, Le QH, Schoen DJ, Bureau TE (2001). Population dynamics of an ac-like transposable element in self- and cross-pollinating Arabidopsis. Genetics.

[70] Lockton S, Gaut BS (2010). The evolution of transposable elements in natural populations of self-fertilizing *Arabidopsis thaliana* and its outcrossing relative *Arabidopsis lyrata*. BMC Evol Biol.

[71] Filatov V, Dowdle J, Smirnoff N, Ford-Lloyd B, Newbury HJ, Macnair MR (2006). Comparison of gene expression in segregating families identifies genes and genomic regions involved in a novel adaptation, zinc hyperaccumulation. Mol Ecol.

[72] Gingeras TR (2007). Origin of phenotypes: genes and transcripts. Genome Res.

[73] López-Maury L, Marguerat S, Bähler J (2008). Tuning gene expression to changing environments: from rapid responses to evolutionary adaptation. Nat Rev Genet.

[74] Heyno E, Klose C, Krieger-Liszkay A (2008). Origin of cadmium-induced reactive oxygen species production: mitochondrial electron transfer versus plasma membrane NADPH oxidase. New Phytologist.

[75] Sarret G, Saumitou-Laprade P, Bert V, Proux O, Hazemann JL, Traverse A, Marcus MA, Manceau A (2002). Forms of zinc accumulated in the hyperaccumulator *Arabidopsis halleri*. Plant Physiol.

[76] Persans MW, Yan X, Patnoe J-MML, Krämer U, Salt DE (1999). Molecular dissection of the role of histidine in nickel hyperaccumulation in *Thlaspi goesingense* (Hálácsy). Plant Physiol.

[77] Tian D, Traw MB, Chen JQ, Kreitman M, Bergelson J (2003). Fitness costs of *R*-gene-mediated resistance in *Arabidopsis thaliana*. Nature.

[78] Bomblies K (2010). Doomed lovers: mechanisms of isolation and incompatibility in plants. Annu Rev Plant Biol.

[79] Bomblies K, Weigel D (2007). Hybrid necrosis: autoimmunity as a potential gene-flow barrier in plant species. Nat Rev Genet.

[80] Lau JA, McCall AC, Davies KF, McKay JK, Wright JW (2008). Herbivores and edaphic factors constrain the realized niche of a native plant. Ecology.

[81] Fine PVA, Mesones I, Coley PD (2004). Herbivores promote habitat specialization by trees in Amazonian forests. Science (New York).

[82] Rajakaruna N (2004). The Edaphic Factor in the Origin of Plant Species. Int Geol Rev.

[83] Borevitz JO, Liang D, Plouffe D, Chang HS, Zhu T, Weigel D, Berry CC, Winzeler E, Chory J (2003). Large-scale identification of single-feature polymorphisms in complex genomes. Genome Res.

[84] Wolfgang H, Poustka A, Vingron M (2002). Variance stabilization applied to microarray data calibration and to the quantification of differential expression. Bioinformatics.

[85] Hubbell E, Liu WM, Mei R (2002). Robust estimators for expression analysis. Bioinformatics (Oxford).

[86] Wu Z, Spencer F, Irizarry RA, Gentleman R, Murillo FM (2004). A model based background adjustment for oligonucleotide expression arrays a model based background adjustment for oligonucleotide expression arrays. J Am Stat Assoc.

[87] Smyth GK (2005). Limma: linear models for microarray data. Bioinformatics and Computational Biology Solutions Using R and Bioconductor.

[88] Suárez-Fariñas M, Pellegrino M, Wittkowski KM, Magnasco MO (2005). Harshlight: a “corrective make-up” program for microarray chips. BMC Bioinforma.

[89] Gautier L, Cope L, Bolstad BM, Irizarry RA (2004). affy–analysis of Affymetrix GeneChip data at the probe level. Bioinformatics.

[90] Benjamini Y, Hochberg Y (1995). Controlling the false discovery rate: a practical and powerful approach to multiple testing. J R Stat Soc Ser B Methodol.

[91] Smyth GK, Speed TP (2003). Normalization of cDNA microarray data. Methods.

[92] Gautier L, Cope L, Bolstad BM, Irizarry RA (2004). affy–analysis of Affymetrix GeneChip data at the probe level. Bioinformatics (Oxford).

[93] SantaLucia J (1998). A unified view of polymer, dumbbell, and oligonucleotide DNA nearest-neighbor thermodynamics. Proc Natl Acad Sci.

[94] Ashburner M, Ball CA, Blake JA, Botstein D, Butler H, Cherry JM, Davis AP, Dolinski K, Dwight SS, Eppig JT, Harris MA, Hill DP, Issel-Tarver L, Kasarskis A, Lewis S, Matese JC, Richardson JE, Ringwald M, Rubin GM, Sherlock G (2000). Gene ontology: tool for the unification of biology. The Gene Ontology Consortium. Nat Genet.

[95] Fisher RA (1922). On the interpretation of *χ*2 from contingency tables, and the calculation of *P*. J R Stat Soc.

[96] Koch MA, Haubold B, Mitchell-Olds T (2000). Comparative evolutionary analysis of *Chalcone Synthase* and *Alcohol Dehydrogenase* loci in Arabidopsis, Arabis, and related genera (Brassicaceae). Mol Biol Evol.

