# Supplementary figures and images for: Between-species differences in gene copy number are enriched among functions critical for adaptive evolution in Arabidopsis halleri
Source: BMC Genomics. 2016 Dec 22;17(Suppl 13):1034. doi: 10.1186/s12864-016-3319-5 (PMC5259951; doi:10.1186/s12864-016-3319-5)

CNEs

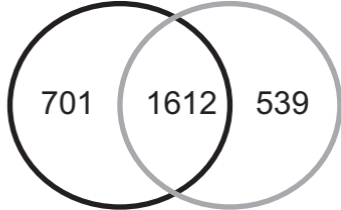

CNRs

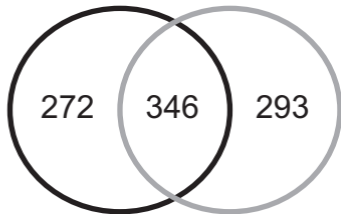

— *A. halleri* hybridizations 1

— *A. halleri* hybridizations 2

Supplement: Additional file 4 — Gene copy number expansions (CNEs) and reductions (CNRs) in A. halleri relative to A. thaliana generated for two datasets, each comprising one of the two A. halleri hybridizations and its in-silico replicate displaying the same amount of within-sample variation as A. lyrata hybridizations. (PDF 23 kb) [file 12864_2016_3319_MOESM4_ESM.pdf]
